# LOCAS – A Low Coverage Assembly Tool for Resequencing Projects

**DOI:** 10.1371/journal.pone.0023455

**Published:** 2011-08-15

**Authors:** Juliane D. Klein, Stephan Ossowski, Korbinian Schneeberger, Detlef Weigel, Daniel H. Huson

**Affiliations:** 1 Chair of Algorithms in Bioinformatics, University of Tübingen, Tübingen, Germany; 2 Genes and Disease Program, Center for Genomic Regulation and Universitat Pompeu Fabra, Barcelona, Spain; 3 Department of Plant Developmental Biology, Max-Planck-Institute for Plant Breeding Research, Köln, Germany; 4 Department of Molecular Biology, Max Planck Institute for Developmental Biology, Tübingen, Germany; University of Georgia, United States of America

## Abstract

**Motivation:**

Next Generation Sequencing (NGS) is a frequently applied approach to detect sequence variations between highly related genomes. Recent large-scale re-sequencing studies as the Human 1000 Genomes Project utilize NGS data of low coverage to afford sequencing of hundreds of individuals. Here, SNPs and micro-indels can be detected by applying an alignment-consensus approach. However, computational methods capable of discovering other variations such as novel insertions or highly diverged sequence from low coverage NGS data are still lacking.

**Results:**

We present LOCAS, a new NGS assembler particularly designed for low coverage assembly of eukaryotic genomes using a mismatch sensitive overlap-layout-consensus approach. LOCAS assembles homologous regions in a homology-guided manner while it performs *de novo* assemblies of insertions and highly polymorphic target regions subsequently to an alignment-consensus approach. LOCAS has been evaluated in homology-guided assembly scenarios with low sequence coverage of *Arabidopsis thaliana* strains sequenced as part of the Arabidopsis 1001 Genomes Project. While assembling the same amount of long insertions as state-of-the-art NGS assemblers, LOCAS showed best results regarding contig size, error rate and runtime.

**Conclusion:**

LOCAS produces excellent results for homology-guided assembly of eukaryotic genomes with short reads and low sequencing depth, and therefore appears to be the assembly tool of choice for the detection of novel sequence variations in this scenario.

## Introduction

Since the introduction of the first Next Generation Sequencing (NGS) technology in 2005, the throughput and cost-efficiency of sequencing has greatly increased and continues to do so. For example, at present, the Illumina HiSeq 2000 yields up to 600 Gb of sequencing data in one paired-end run taking about one week. While the accuracy of new sequencing technologies is similar to that of Sanger sequencing, the achievable read length has decreased from 1 kb to around 150 bases or less for Illumina's GAIIx or HiSeq and Applied Biosystem's SOLiD instruments, or to less than 500 bases for the GS FLX Titanium instrument from Roche/454 Life Sciences.

NGS technologies have many different applications, including genome sequencing and resequencing, metagenome analysis, transcriptome analysis and chromatin immunoprecipitation (ChIP)-sequencing. To afford genome sequencing of hundreds to thousands of individuals large-scale re-sequencing projects like the Human 1000 Genomes Project utilize low coverage sequencing to a depth of less than 5× [1], followed by mapping of reads to a known reference genome from the same species. This *alignment-consensus* approach, used by e.g. SOAPsnp [2], VAAL [3], MAQ [4], Pyrobayes [5], SHORE [6] or SHRiMP [7], is capable to detect sequence variants like single-nucleotide polymorphisms (*SNPs*) or small insertions or deletions (micro-*indels*) [8], [9]. However, regions with high divergence will not be discovered with the alignment-consensus approach since the respective reads are mostly unalignable to the reference genome. Various approaches to estimate copy number variants and other large rearrangements (commonly referred to as structural variants) [4], [6], [10] from read quantity or mate-pair data have been introduced but these strategies do not reveal additional sequence information.

As *de novo* assembly does not rely on read alignments against a reference genome, it appears to be better suited for studying unknown or highly diverged regions. Unfortunately, *de novo* assembly of short reads still poses unsolved issues for eukaryotic genomes. Here, the size and complexity of the genomes place high demands on computational power, which cannot be met by standard servers. Typically, such assemblies also suffer from a high number of short contigs. To cope with these problems, several strategies have been proposed, e.g. reduced representation libraries [10], gene-boosted assembly [11], transcriptome assembly [12] or homology-guided assembly [13].

In contrast to *de novo* assembly, *homology-guided* (or comparative) assembly approaches are more appropriate to discover novel sequence regions in eukaryotic genomes. Like the alignment-consensus approach, homology-guided assembly approaches exploit available reference genome assemblies from the same or closely related species. One strategy to perform homology-guided assembly starts with aligning short reads to a reference followed by local assemblies of reads that are aligned within the same regions (called *blocks*). We will refer to local assemblies based on alignment information within a block as *reassembly*.

Reassembly can also benefit from incorporating reads that cannot be aligned (i.e. *left-over* reads) as they often originate from insertions or highly polymorphic regions, and therefore can potentially elongate the reassembled sequence on either side. Currently available assembly tools do not provide time-efficient methods to incorporate left-over reads for consecutive execution of multiple reassemblies. The set of left-over reads can be huge compared to the set of reads belonging to one block of the sequenced genome. Left-over reads consist not only of reads from highly polymorphic regions but also of erroneous reads, which, in our experience, can comprise about 5% to 20% of all reads from a sample. Since existing assemblers do not distinguish between aligned reads and left-over reads, they would have to assemble all left-over reads for each block over and over, leading to an unacceptable increase in runtime.

Genome resequencing is often performed at low read coverage or *sequencing depth*. This results from a simple cost-benefit analysis: even with a sequencing depth of 7×, most of the reference genome is covered by aligned reads, enabling the detection of most SNPs and small indels. However existing short read assemblers based on the de Bruijn graph paradigm such as VELVET [14], EULER-SR [15] and ABySS [16] are not well suited for low sequencing depth assembly as they typically require coverages of 20× to 30× [14]. Furthermore, de Bruijn graph assemblers rely on exact matches of sub-regions (kmers) of reads and do not calculate overlap alignments. Consequently, they cannot detect overlaps of reads with a substantial number of mismatches. For low sequencing depth assembly, however, it is necessary to include as many overlaps as possible in order to assemble low-coverage regions.

We have developed a new assembly tool, LOCAS (LOw Coverage Assembly Software), with two main goals in mind: 1) allowing for *de novo* assembly at low sequence coverage, 2) support whole genome homology-guided assembly approaches by incorporating left-over reads. Thus LOCAS is designed for assembling short to medium sized reads either *de novo* or in a homology-guided fashion using an overlap-layout-consensus approach. It explicitly handles data of low sequencing depth by allowing mismatches in the overlap calculation of reads. Further, to improve homology-guided assembly, it optionally takes advantage of the given alignment positions of reads relative to a reference sequence. An extension of LOCAS, called SUPERLOCAS, efficiently handles left-over reads. It calculates an overlap graph for the left-over reads once and recruits relevant overlapping parts of this graph during each reassembly quickly and efficiently. We show that LOCAS and SUPERLOCAS produce assemblies that are often better than those obtained by existing short read assemblers at sequencing depth of 7.5× as measured by N50 size and error rate.

## Methods

LOCAS is based on the classical overlap-layout-consensus approach. We extended the approach described by Kececioglu and Myers [17], [18], which was originally developed for assembling Sanger reads. After calculating an approximation of all alignments between all reads, an overlap graph is built. The graph is reduced and transformed into a path graph in which the vertices represent the unique paths of the overlap graph. Contigs are finally generated by extraction of appropriate paths from the path graph. To adjust this approach to short-read datasets of low sequencing depth, we modified the algorithm that selects the final paths in the path graph to handle a higher amount of single reads and a shorter read length. The higher amount of reads increases the complexity of the graph. Moreover, the shorter read lengths lead to shorter overlap alignments, also contributing to the complexity of the graph. In addition, more false overlap alignments are found for a shorter alignment length. We modified the algorithm to handle the increased number of false alignments and the larger graph as well as the relative increase of branches in the graph. Paths are selected in the path graph using a greedy strategy that aims at maximizing the total coverage of used reads and the total quality of the alignments used.

### Overlap Detection and Overlap Alignment Graph

Pairs of potentially overlapping reads are detected with the help of an enhanced suffix array as implemented by the SeqAn library [19]. The suffix array is built for all reads followed by identification of all pairs of reads sharing an identical kmer. Overlap alignments are calculated for each pair with a minimal overlap length and a maximal number of mismatches given as input parameters to the assembly tool. The alignment information is represented by an overlap graph.

Each vertex of the graph represents a read and each edge represents an alignment between the reads of the connected vertices. Two types of edge exist. An overlap edge corresponds to an overlap alignment. A containment edge represents a global alignment. Containment edges indicate that one read is aligned over its full length to the other read. If the reads are not equally long, then one read will be contained. A weight is assigned to each edge that corresponds to the length of the alignment. Further, a score is given that is equal to the alignment score describing the quality of the alignment. In the next step, an exemplar vertex is chosen for each set of vertices that are all connected to each other by containment edges. The exemplar is the longest read with the highest alignment score to the other reads in the set. For all other vertices represented by the exemplar, all overlap edges are removed. See Figure S3 for an illustration of the reduction.

### Reduction and Path Graph

**Figure 1:**
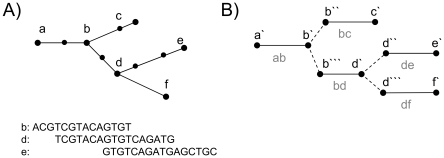
Transformation of the overlap alignment graph into the path graph. In (A), an overlap graph is shown, and, for some read sequences, the underlying alignment information is displayed. In (B), the corresponding path graph is displayed. For each unique path in the overlap graph an inner edge is introduced in the path graph, represented by a solid line. For example, the unique path between b and d in the overlap graph is represented by the inner edge (b′″, d′) in the path graph. If two vertices in the path graph represent the two ends of the same read, then a real edge is presented between them in the path graph. These real edges are shown as dashed lines. An example is the real edge (d′, d″). The vertices d′ and d″ represent the same read since they both correspond to vertex d in the overlap graph but they represent the two different ends of the read, since the read of d overlaps with the read of b and e but at different ends, which is displayed in (A).

The overlap graph is reduced by removing all transitive edges. Each edge e_t_ = (v_1_, v_2_) with a weight w_t_ is transitive if two edges e_1_ = (v_1_, w) and e_2_ = (w, v_2_) exist with weights w_1_ and w_2_, respectively, where w_t_≤w_1_ and w_t_≤w_2_. See Figure S4 for an illustration. Unique paths in the overlap graph are detected and merged. This is done during a transformation of the overlap graph into a path graph. Each unique path of maximal length in the overlap graph is represented by two vertices and an inner edge in the path graph. The two vertices in the path graph represent the ends of the path or, more precisely, the outer ends of the first and the last read sequence on the path. Further, we insert real edges that represent the adjacencies between unique paths in the overlap graph. A real edge is set for two vertices in the path graph if these vertices represent different ends of the same read. This is illustrated in Figure 1.

### Solving Sequencing Errors and Repeat Structures

Sequencing errors and repeat structures correspond to cycles in the path graph. They are called *directed cycles* if they represent repeat structures and *undirected cycles* if the represent sequencing errors or false alignments (Figure S5). An algorithm is applied to the path graph that calculates a spanning-treelike subgraph. The algorithm works as follows. First, all real edges are placed according to their score in a priority queue. This edge score measures the average quality of the read alignments and the average read coverage from reads underlying the edge vertices. The edges of the subgraph are selected iteratively in the path graph. In each step, the edge with the highest score is considered for selection. If a directed cycle will not be introduced in the subgraph if a certain edge were selected, the edge is selected. If the candidate edge would introduce a directed cycle in the subgraph, the edge is not selected, the cycle is cleaned of all short dangling paths and then cut open at all remaining branch vertices. Next, the remains of the opened directed cycle are recorded with its branches, called repeat branches. See Figure S6 for an illustration of the cutting operation.

If an undirected cycle with a similar sequence is introduced with the selection of the candidate edge, then the edge is selected and the whole cycle is marked in the subgraph. For the introduction of an undirected cycle with a dissimilar sequence, the edge is not selected. It is likely that some alignments are not detected due to sequencing errors. In this case, the affected repeats are not represented by directed cycles in the spanning-treelike subgraph. In order to detect and solve these repeat structures, the algorithm scans for fragmentary directed cycles. They correspond to paths in the graph that link two branch vertices so that each of them is adjacent to at least one other short path. These branch vertices are denoted as repeat branches, as in the case of directed cycles. The repeat branches are detected in the spanning-treelike subgraph and their real edges are cut.

With mate-pair information, it is possible to assign two repeat branches to each other. In this case, the path in between can be duplicated and re-linked in accordance with the mate-pair information. Here, our strategy is similar to the one described by Pevzner and Tang [20].

### Path Extraction and Consensus Determination

All the longest paths are calculated for the given spanning-treelike subgraph. For each leaf in the subgraph, the longest path is determined. For the end-vertices of these longest paths, the longest paths are again calculated. Then the resulting set of longest paths is scanned for paths that share a subpath with each other. For these paths, only the longest path is taken for the final set. Finally, the consensus is determined for each path in the final set of longest paths considering all overlap alignments and global alignments to contained reads.

### Algorithm to Integrate Left-Over Reads

**Figure 2:**
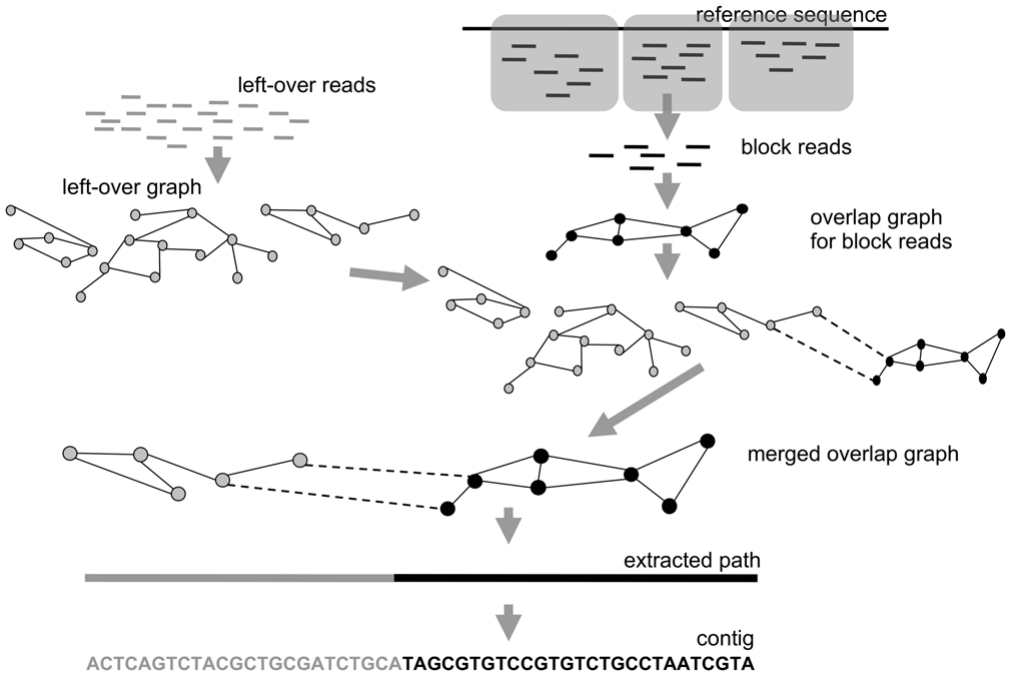
Workflow of SUPERLOCAS. The figure shows the workflow of the algorithm of SUPERLOCAS. The initial steps are illustrated: the left-over reads with the constructed left-over overlap graph, and the reads that are aligned against the reference sequence and partitioned into blocks. Next, the steps that are executed consecutively for each block are shown: the construction of the overlap graph, the insertion of edges between both graphs and the procedure until contigs are reported for the merged graph.

SUPERLOCAS is an extension of LOCAS that explicitly takes left-over reads into account when using LOCAS for local or homology-guided assemblies (Figure 2). First, an overlap graph for all left-over reads is built, called the left-over graph. Then, a modified version of the basic assembly algorithm is applied to each block of the genome, which also deals with the integration of left-over reads. The extra step is applied after having built the overlap graph: first, the relevant left-over reads are selected by determining overlap alignments between local reads and left-over reads. For the selected left-over reads, the induced subgraph of the left-over graph is linked to the overlap graph of the local assembly. By extending the overlap graph with parts of the left-over graph, left-over reads are taken into account during the following assembly steps and can substantially elongate the resulting contigs.

## Results

### Assembly Algorithm and Extension for Homology-Guided Assemblies

We have developed and implemented a new assembly algorithm in a software tool called LOCAS. The approach is based on the overlap-layout-consensus paradigm. Especially designed for data of low sequencing depth in the context of resequencing projects, LOCAS calculates alignment based overlaps instead of exact matches between reads.

Furthermore, we have implemented an extension to LOCAS called SUPERLOCAS to support our homology-guided assembly approach. In this approach, the target genome is partitioned into blocks of given length. Next, SUPERLOCAS assembles each set of reads aligning one of the blocks separately by incorporating high quality left-over reads in the assembly. Thus, the tool can assemble very long insertions of several kb located between two highly homologous regions. In order to reduce the complexity of the overlap graph, different alignment constraints can be set as a function of the distance between reads in the reference genome.

### Performance Evaluation

We evaluated the performance of LOCAS in three studies. The first study simulated *de novo* assemblies of small genomic regions and compared the performance of current NGS assemblers for data with a low sequencing depth. For the second study, we simulated a homology-guided assembly of a divergent strain of *Arabidopsis thaliana*, again at low sequencing depth. For the third study, we performed a homology-guided assembly using Illumina reads from the *Arabidopsis thaliana* 1001 Genomes Project (http://1001genomes.org/). For the first study, we compared LOCAS with the publicly available short read assembly tools VELVET [14], EULER-SR [15], ABySS [16], [21] and SOAPdenovo [22]. In the second and third studies, we compared SUPERLOCAS (LOCAS extension for homology-guided assembly) only with VELVET, since the other assemblers (ABySS, SOAPdenovo and EULER-SR) did not perform well enough considering contig length and error rate in the first study. Even though EDENA [23] also uses an overlap-layout-consensus approach, we did not consider it in our evaluation, because it does not utilize paired read information, does not allow for mismatches in the overlap calculation and produced insufficient results for low sequencing depth assemblies.

Homology-guided assemblers like AMOScmp-shortReads (unpublished) and its predecessor AMOScmp [24] provide an integrated solution using alignments to the reference in both the overlap as well as the consensus and scaffolding step, while LOCAS relies on pre-computed alignments from third party tools and does not use homology information in the consensus or scaffolding step of the assembly. Thus resulting contigs are hardly comparable without further post-processing as for instance implemented in the SHORE homology-guided assembly framework [25] combining LOCAS and other short read assemblers with the scaffolding tool BAMBUS [26] and quality assessment based on re-alignments of original reads to novel contigs.

All datasets used for evaluation are available at http://www-ab.informatik.uni-tuebingen.de/software/locas.

### Evaluation of Low Sequencing Depth Assembly

We used the first chromosome of *A. thaliana* Col-0 for our performance studies. First, we simulated Illumina GAIIx reads at a sequencing depth of 7.5× using METASIM [21]. We chose this particular sequencing depth since it is the lowest in which the assemblers showed reasonable contig sizes. The reads were assigned to reference sequence corresponding to their origin positions and partitioned into blocks of 10 kb length. Assemblies of the blocks were performed separately using the assembly tools LOCAS, ABySS, EULER-SR, VELVET and SOAPdenovo.

Besides other measures (see Methods), we primarily compared the N50 size averaged over all local assemblies, denoted as *avgN50*, and the average error rate of the contigs, denoted as *avgERR*. In our study, the N50 size is defined as the length of the longest contig such that all contigs of equal or longer length cover at least 50% of the positions of the block sequence. For the calculation of avgN50, we considered only *valid* contigs that have a global alignment to the target sequence with at most 10% mismatches. The error rate is the total number of errors divided by total length of all contigs. The total number of errors comprises the number of mismatches in the alignment of all valid contigs plus the lengths of other contigs.

We ran all assemblers using a wide range of parameter settings to show the achievable avgN50 and avgERR values with the respective assembler (see Table S1). This is a common approach to optimize the assembly. For example the method is implemented in VelvetOptimizer to be used for VELVET.

**Figure 3:**
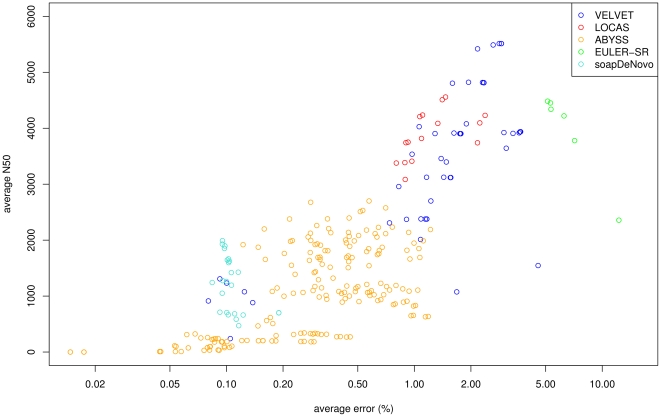
Performance comparison of low sequencing depth assembly. Illumina GAIIx reads were simulated at a sequencing depth of 7.5× for the first chromosome of *A. thaliana* Col-0. The reads were assigned to the reference sequence corresponding to their origin positions and partitioned into blocks of a length of 10 kb. The avgN50 (average N50) is plotted against the avgERR (average error rate) for the assembly tools LOCAS, EULER-SR, ABySS, VELVET and soapDeNovo. For each assembler, several runs are displayed corresponding to the different parameter settings. The data points of ABySS are drawn in orange, EULER-SR in green, LOCAS in red, VELVET in blue and soapDeNovo in turquoise. Each point represents one run.

The results are shown in Figure 3. For avgERR values lower than 1.5%, LOCAS performed best with a maximum avgN50 size of 4,558 bp. For an avgERR higher than 1.5%, VELVET performed best with a maximum avgN50 size of 5,500 bp. EULER-SR performed well with respect to avgN50 size, but had a high avgERR of at least 5%. The avgERR values of ABySS were with at most 1.2% very low, but the tool showed an avgN50 size of at most 2,577 bp. The avgERR values were even lower for SOAPdenovo, while its avgN50 size was the lowest for all assemblers with a maximum of 1,991 bp. We also examined CPU time and RAM needed to assemble the whole data set for each tool. The best performing method regarding CPU time was VELVET with 10 min in average, followed by LOCAS with 19 min, SOAPdenovo with 22 min and EULER-SR with 140 min. LOCAS and EULER-SR used only 18 MB of RAM, VELVET used 87 MB and SOAPdenovo used 236 MB. (See Figure S1 and S2 for results of this performance study with a sequencing depth of 5× and for the fourth chromosome, respectively.)

### Evaluation of Homology-Guided Assembly

To evaluate performance in a homology-guided assembly approach, we simulated a resequencing study of an artificial *A. thaliana* strain using a sequencing depth of 7.5×. First, we artificially generated a target genome by introducing SNPs, insertions and deletions into the reference sequence (see Text S1). Then we used this target genome to simulate Illumina reads similar to the previous study. Reads were aligned to the reference genome and partitioned into blocks of 25 kb length using SHORE [6], a short read analysis framework supporting several alignment tools like BWA [27], Bowtie [28] and GenomeMapper. For this study, we preferred GenomeMapper [29] to the other tools because it allows for high edit distances including gaps and has a high sensitivity thus improving homology information generated by read alignments in highly diverged regions.

Next, all left-over reads were pooled. Assemblies of the blocks incorporating the left-over reads were performed applying the assembly tools SUPERLOCAS and VELVET. As VELVET does not provide a special mode for left-over incorporation, we used VELVET as follows: each local assembly was given the complete set of left-over reads as an additional input. We omitted EULER-SR, SOAPdenovo and ABYSS due to their insufficient performance shown in the first study on data with a low sequencing depth.

**Figure 4:**
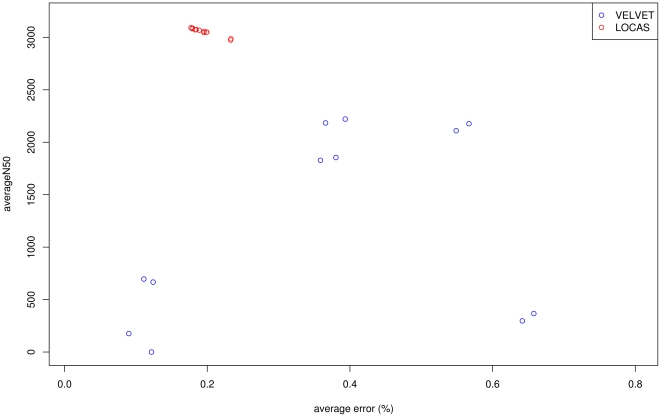
Performance comparison of homology-guided assembly on simulated data. We simulated a resequencing study of an artificial *A. thaliana* strain using a sequencing depth of 7.5×. The simulated Illumina reads were aligned to the reference genome Col-0 and partitioned into blocks of 25 kb using SHORE. The assembly tools SUPERLOCAS and VELVET were applied to assemble the mapped reads of the first chromosome and the left-over reads. The avgN50 (average N50) is plotted against the avgERR (average error rate) for the assembly tools SUPERLOCAS and VELVET (in left-over incorporation mode). SUPERLOCAS is displayed in red and VELVET in blue.

In Figure 4, the resulting avgN50 size and avgERR are shown. SUPERLOCAS performed robustly with different parameters and overall outperformed VELVET in our study. We observed a maximum avgN50 size of 3,132 bp and 2,446 bp for SUPERLOCAS and VELVET, respectively. The error rates range from 0.17% to 0.21% for SUPERLOCAS, and from 0.09% to 0.53% for VELVET. The average maximal contig size of SUPERLOCAS was larger (with up to 17,821 bp) in comparison with VELVET (up to 12,996 bp). (See Table S2 for more details.) The CPU runtimes ranged from 3 h 12 min for SUPERLOCAS to 7 h 51 min for VELVET per run. SUPERLOCAS used 433 MB of RAM while VELVET required 224 MB.

**Figure 5:**
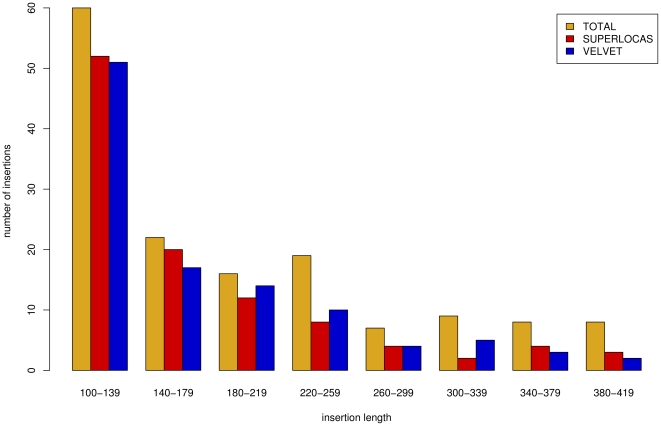
Number of detected insertion regions in homology-guided assembly on simulated data. For the artificial *A. thaliana* strain in the simulated resequencing study, the total insertion regions in the target genome are plotted for different lengths of these regions. In addition, the number of error-free regions assembled by VELVET and by SUPERLOCAS are shown.

In addition, we examined the contigs of both tools regarding the appearance of insertion regions. These regions are present in the target genome but not in the reference genome. Most insertion regions with a length of at least 100 bp could only be assembled with the help of left-over reads. Figure 5 shows the number of insertion regions that were assembled without errors by SUPERLOCAS and by VELVET, respectively, separated by insertion length. Both tools performed this task equally well, assembling a similar number of the insertions.

### Real World Data

To test performance on real world data, we used sequence reads from the first chromosome of *A. thaliana* strain Landsberg erecta (Ler-1) produced within the 1001 Genomes Project [30]. Ler-1 was sequenced on the Illumina GAIIx with 80 bp paired-end reads to a depth of ∼7×. Reads were aligned against the complete reference sequence and all alignments on chromosome 1 have been partitioned into subregions of at most 40 kb using GenomeMapper [29] and SHORE [6]. We first applied LOCAS and VELVET to the assembly of the reads in each block without left-over reads. Instead of the avgERR, we estimated the average relative dissimilarity to the reference sequence over all blocks, denoted as *avgDISS*.

**Figure 6:**
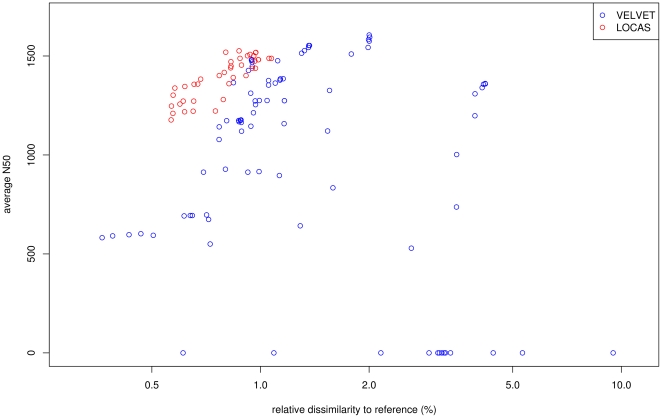
Performance comparison of homology-guided assembly on real world data without utilizing left-over reads. Paired-end reads were produced by Illumina GAIIx with a length of 80 bp to a depth of ∼7× for the first chromosome of *A. thaliana* strain Ler-1. Reads were aligned against the complete reference sequence (Col-0) and partitioned into blocks with SHORE of 25 kb. LOCAS and VELVET are applied in paired-end mode for all blocks which contain reads that are aligned to the same region of the reference sequence. The x-axis shows the avgN50 (average N50) and the y-axis the avgDISS (average dissimilarity). The runs of LOCAS produced with different parameter setting are drawn in red and those of VELVET in blue.

For avgDISS values higher than 1%, VELVET performed best considering avgN50 size, while for avgDISS values lower than 1%, LOCAS showed the best avgN50 sizes (Fig. 6). We observed a maximum avgN50 size of 1,606 bp and of 1,526 bp for VELVET and LOCAS, respectively.

**Figure 7:**
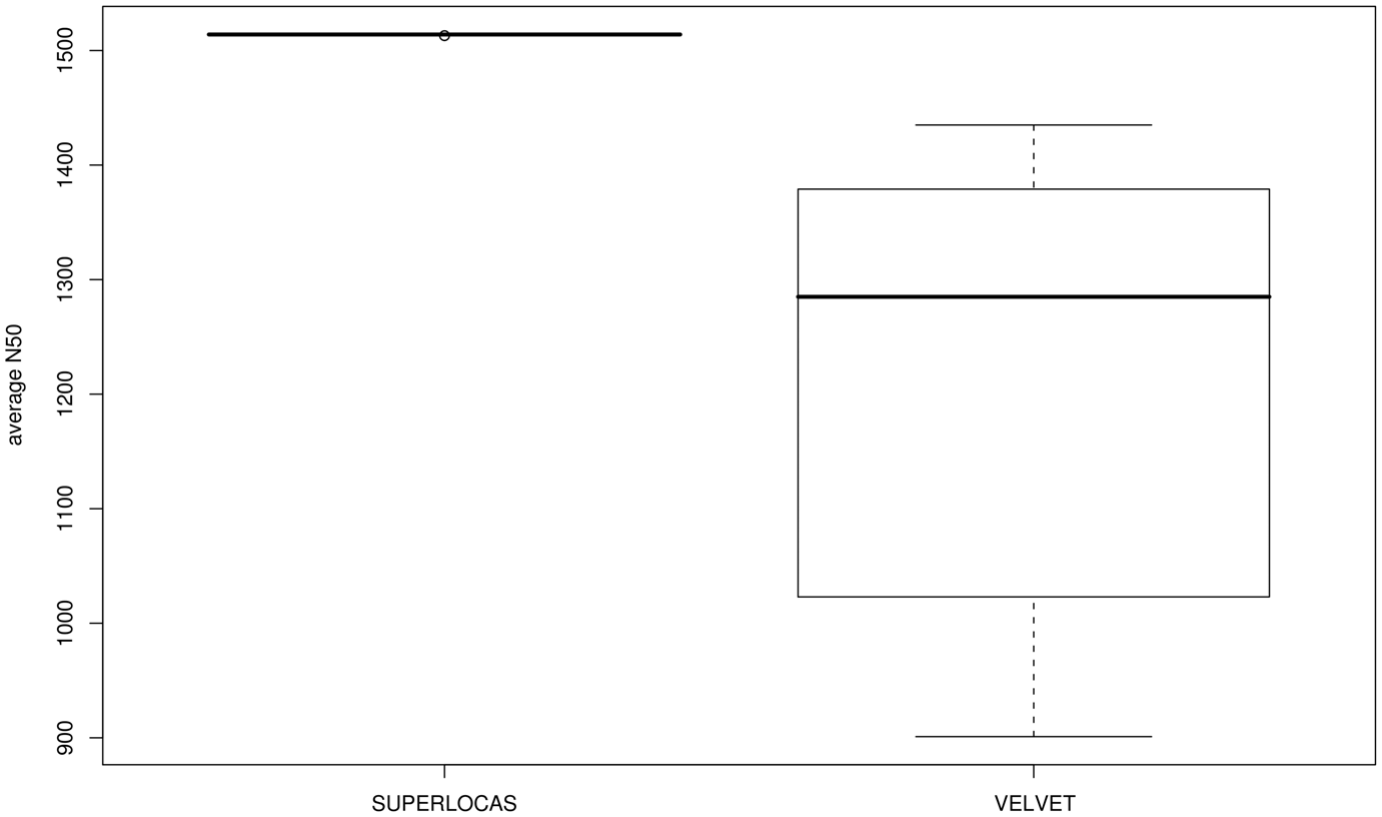
Performance comparison of homology-guided assembly on real world data utilizing left-over reads. Illumina reads of the first chromosome of *A. thaliana* strain Ler-1 were aligned against the reference sequence (Col-0) and partitioned into blocks with SHORE of 25 kb. Local assemblies of reads are performed with SUPERLOCAS and VELVET in order to incorporate left-over reads. While SUPERLOCAS provides algorithms specifically adjusted to this task, VELVET had to assemble each block with the complete set of left-over reads. A barplot is shown for the avgN50 (average N50) size of both assemblers.

We then applied SUPERLOCAS and VELVET. For VELVET, each block was assembled with the complete set of left-over reads as in the second study. Contigs were used for analysis if they featured a similarity with the reference sequence of at least 75%. We allowed this high percentage of dissimilarity since contigs that are constructed with the use of left-over reads often belong to insertion regions not represented in the reference genome. N50 sizes were higher for SUPERLOCAS with values consistently about 1500 bp (Figure 7). N50 sizes of VELVET ranged between 901 bp and 1,435 bp, showing much higher sensitivity to parameter choice. Furthermore, SUPERLOCAS performed best regarding CPU runtime. For SUPERLOCAS, one run was on average completed in 2 h 8 min, compared to 7 h 32 min for VELVET. VELVET performed best considering RAM usage. SUPERLOCAS used 3.99 GB of RAM while VELVET used only 1.73 GB. (See Table S3 and S4 for more details.).

## Discussion

The assembly tool LOCAS is specifically designed for homology-guided assembly approaches in resequencing studies of eukaryotic genomes with short reads and low sequencing depth. In three performance studies adjusted to these conditions, LOCAS achieved usually better or at least similar results compared to existing short read assemblers. SUPERLOCAS, an extension of LOCAS, proved to be much faster than existing methods for the task of integrating left-over reads in consecutive executions of local reassemblies. The performance of SUPERLOCAS at low sequencing depth as measured by avgN50 and avgERR compares favorably to all other tools.

LOCAS uses the overlap-layout-consensus approach because we found it to be better suited for low sequencing depth assembly in comparison to the de Bruijn paradigm used in VELVET, EULER-SR and ABYSS. We optimized overlap alignments between reads to span even very low covered regions. However, the number of overlaps increases by calculating exact alignments instead of submatches and often leads to a graph of higher complexity compared to a Bruijn graph. Moreover the graph cannot be reduced by using a coverage-based cutoff as typically applied in de Bruijn graph approaches, since the insufficient number of reads in low coverage assembly prevents the estimation of a reasonable value for a coverage-based cutoff. Further, the probability of using false overlaps increases for alignment calculations that allow for several mismatches compared to exact sub-matches, and the calculation of the overlap graph is slower than the construction of the de Bruijn graph. Nevertheless, longer contigs and a higher overall coverage of the sequenced genome are the payoff for dealing with a more complex graph and an increase in false alignments. In scenarios of high sequence coverage, LOCAS often produces shorter contigs compared to de Bruijn graph based *de novo* assemblers, which is a consequence of the highly sensitive overlap detection of LOCAS and the resulting overlap-graph of higher complexity.We believe that the method implemented in SUPERLOCAS is a good compromise between resource heavy *de novo* assembly and simple homology-guided assembly approaches that do not incorporate left-over reads. The integration of left-over reads allows for identification of polymorphic or inserted regions in a *de novo* assembly fashion. Additionally, our assembly approach makes use of the exact positions of aligned reads against a reference in order to reduce assembly complexity and to reduce the number of false overlaps, as was formerly used to improve overlap calculation of Sanger reads [31]. Further, we speculate that the assembly produced by our approach might be less affected by repetitive regions than *de novo* assembly because some repeats are already solved during the alignment step. Since SUPERLOCAS explicitly treats left-over reads and aligned reads differently, and can reuse the left-over graph in multiple local assembly executions, the incorporation of left-over reads is more reliable and much faster than for other short read assemblers.

We assume that in future, our method will gain from improvements in sequencing technologies. Our overlap-layout-consensus approach can benefit from longer reads as it is more robust to sequencing errors at the end of reads. Computing overlap alignments rather than using exact matches of sub-regions (like de Bruijn graph methods do) in order to overlap the reads will become more important as longer sub-matches have a higher probability of containing sequencing errors. In addition, the recruitment of left-over reads will become more reliable due to an increased overlap length between reads. Finally, the potential of homology-guided assemblies grows steadily with increasing numbers of completely sequenced genomes.

### Design and Implementation

The software is implemented in C++ and supported on Linux.

### Availability

Source code and binaries are freely available from our website http://www-ab.informatik.uni-tuebingen.de/software/locas.

## Supporting Information

Figure S1 **Performance comparison of assembly with a sequencing depth of 5× for the first chromosome of** ***A. thaliana*** **.** Illumina reads with a sequencing depth of 5× were simulated for the first chromosome of *A. thaliana* Col-0. The reads were assigned to the reference sequence corresponding to their origin positions and partitioned into blocks of a length of 10 kb. The avgN50 (average N50) is plotted against the avgERR (average error rate) for the assembly tools LOCAS, EULER-SR, ABySS, VELVET and SOAPdenovo. For each assembler, several runs are displayed corresponding to the different parameter settings. The data points of ABySS are drawn in orange, EULER-SR in green, LOCAS in red, VELVET in blue and SOAPdenovo in turquoise.
(DOC)

Figure S2 **Performance comparison of assembly with a sequencing depth of 7.5× for the fourth chromosome of** ***A. thaliana*** **.** Illumina reads were simulated for the fourth chromosome of *A. thaliana* Col-0 with a sequencing depth of 7.5×. After the reads were assigned to their origin positions in the reference sequence they were partitioned into blocks of a length of 10 kb. The avgN50 (average N50) is plotted against the avgERR (average error rate) for the assembly tools LOCAS, EULER-SR, ABySS, VELVET and SOAPdenovo. For each assembler, several runs are displayed corresponding to the different parameter settings and ech data point corresponds to such a run. The data points of ABySS are drawn in orange, EULER-SR in green, LOCAS in red, VELVET in blue and SOAPdenovo in turquoise.
(DOC)

Figure S3 **Construction of the overlap graph.** Eight aligned reads are displayed on the left. Each read has an alignment to all other reads. Most alignments are overlap alignments, while the alignments among the reads 1 to 3 and 4 to 7 are global alignments. The alignment graph for the aligned reads is shown on the right. For each read, a vertex is introduced into the graph; for each overlap alignment, an overlap edge is introduced; for each global alignment, a containment edge. Reads 1 and 4 are chosen as exemplar vertices. For all other reads, all edges are deleted except those leading to the exemplars. The deleted edges are shown as dashed lines; solid lines indicate the edges that have been retained.
(DOC)

Figure S4 **Transitive reduction of the overlap graph.** The edge (v, z) is transitive since two edges (v, w) and (w, z) exist and their weights less than or equal to the weight of (v, z). A transitive edge is then reduced.
(DOC)

Figure S5 **Different cycle types in the path graph.** (A) An example of an undirected cycle is shown. If the consensus sequences of both cycle branches are similar, the structure arises from a sequencing error or an SNP. If the consensus sequences of both branches are dissimilar a false alignment could be the reason. (B) A directed cycle that usually arises from repeats is shown. (C) A directed cycle that is connected with an undirected cycle is shown. Such a structure can arise from several repeat copies.
(DOC)

Figure S6 **Cutting a directed cycle in the path graph.** (A) A directed cycle with short dangling paths is displayed. (B) The cycle is shown after all short paths have been cut off and the cycle has been cut open at each branch vertex.
(DOC)

Table S1 **Evaluation results of low sequencing depth assembly with LOCAS, VELVET, EULER-SR and ABySS.**
(DOC)

Table S2 **Evaluation of homology-guided assembly on simulated data with SUPERLOCAS and VELVET.**
(DOC)

Table S3 **Evaluation of homology-guided assembly on real world data with LOCAS and VELVET (without utilizing left-over reads).**
(DOC)

Table S4 **Evaluation of homology-guided assembly on real world data with SUPERLOCAS and VELVET (utilizing left-over reads).**
(DOC)

Text S1 **Supplementary Results and Methods.** Results of further assemblies for the first chromosome of A. *thaliana* at a sequencing depth of 5× and for the fourth chromosome of A. *thaliana* at a sequencing depth of 7× are shown. In addition, the usage of LOCAS and SUPERLOCAS is described. In the Section Methods the read simulation method, the simulation of a homology-guided assembly and the assembly analysis are described more precisely.
(DOC)
