# Supplementary material for: LOCAS – A Low Coverage Assembly Tool for Resequencing Projects
Source: PLoS One. 2011 Aug 15;6(8):e23455. doi: 10.1371/journal.pone.0023455 (PMC3156226; doi:10.1371/journal.pone.0023455)
Supplement: Table S1 — Evaluation results of low sequencing depth assembly with LOCAS, VELVET, EULER-SR and ABySS. (DOC) [file pone.0023455.s007.doc]

Table S1A - Evaluation of low sequencing depth assembly with LOCAS.

| **Parameter Settings** | **Mean** | **Min** | **Max** | **N50** | **N75** | **N90** | **Coverage** | **Error** | **Total Error** | **Unmapped** | **All** |
| --- | --- | --- | --- | --- | --- | --- | --- | --- | --- | --- | --- |
| kmer:13 -L 13 -S 4 | 1655.55 | 180 | 6481 | 4230 | 2758 | 1179 | 0.978185 | 0.003291 | 0.023799 | 493 | 21601 |
| kmer:13 -L 15 -S 4 | 1774.28 | 192 | 6739 | 4558 | 2939 | 1257 | 0.97988 | 0.00305 | 0.014654 | 279 | 21419 |
| kmer:13 -L 17 -S 4 | 1680.32 | 174 | 6432 | 4236 | 2674 | 1172 | 0.981362 | 0.002954 | 0.011056 | 196 | 21364 |
| kmer:13 -L 19 -S 4 | 1541.52 | 159 | 6029 | 3752 | 2312 | 1019 | 0.981487 | 0.002884 | 0.009258 | 153 | 21327 |
| kmer:13 -L 21 -S 4 | 1429.91 | 155 | 5668 | 3386 | 2086 | 927 | 0.980997 | 0.00287 | 0.008904 | 144 | 21301 |
| kmer:13 -L 13 -S 2 | 1281.09 | 137 | 6084 | 3740 | 2345 | 948 | 0.983857 | 0.00377 | 0.021682 | 474 | 23269 |
| kmer:13 -L 13 -S 3 | 1554.05 | 167 | 6404 | 4096 | 2641 | 1113 | 0.981248 | 0.003365 | 0.022303 | 470 | 22018 |
| kmer:13 -L 15 -S 2 | 1455.62 | 140 | 6345 | 4087 | 2568 | 1121 | 0.983368 | 0.003374 | 0.013336 | 262 | 22589 |
| kmer:13 -L 15 -S 3 | 1726.75 | 183 | 6699 | 4510 | 2893 | 1264 | 0.98089 | 0.003062 | 0.014079 | 271 | 21663 |
| kmer:13 -L 17 -S 2 | 1428.57 | 137 | 6083 | 3818 | 2414 | 1062 | 0.982809 | 0.003145 | 0.010918 | 201 | 22302 |
| kmer:13 -L 17 -S 3 | 1650.79 | 172 | 6415 | 4208 | 2648 | 1175 | 0.981419 | 0.002904 | 0.010691 | 190 | 21530 |
| kmer:13 -L 19 -S 2 | 1326.28 | 131 | 5711 | 3411 | 2093 | 926 | 0.981576 | 0.002986 | 0.0097 | 169 | 22096 |
| kmer:13 -L 19 -S 3 | 1516.14 | 158 | 6009 | 3742 | 2295 | 1012 | 0.981554 | 0.002783 | 0.009015 | 150 | 21469 |
| kmer:13 -L 21 -S 2 | 1247.87 | 130 | 5378 | 3085 | 1907 | 842 | 0.982272 | 0.002952 | 0.00894 | 148 | 22027 |
| kmer:13 -L 21 -S 3 | 1411.73 | 153 | 5647 | 3378 | 2079 | 926 | 0.981649 | 0.002762 | 0.008017 | 127 | 21440 |

Table S1B - Evaluation of low sequencing depth assembly with VELVET.

Parameter settings: -ins_length 300 -ins_length_sd 30 -scaffolding no –shortPaired

| **Parameter Settings** | **Mean** | **Min** | **Max** | **N50** | **N75** | **N90** | **Coverage** | **Error** | **Total Error** | **Unmapped** | **All** |
| --- | --- | --- | --- | --- | --- | --- | --- | --- | --- | --- | --- |
| kmer: 13 -exp_cov 5 | 598.52 | 105 | 3046 | 1075 | 483 | 86 | 0.861407 | 0.00366209 | 0.0168096 | 242 | 17944 |
| kmer: 13 -exp_cov 9 | 2202.04 | 262 | 6216 | 3908 | 2383 | 859 | 0.897908 | 0.00698525 | 0.0335045 | 508 | 18429 |
| kmer: 13 -exp_cov 15 | 2241.03 | 271 | 6247 | 3938 | 2402 | 864 | 0.896879 | 0.0074156 | 0.0367096 | 560 | 18409 |
| kmer: 13 -exp_cov 19 | 2241.65 | 272 | 6247 | 3938 | 2402 | 864 | 0.89696 | 0.0074251 | 0.0367827 | 561 | 18411 |
| kmer: 15 -exp_cov 5 | 1501.26 | 139 | 5819 | 3459 | 2015 | 609 | 0.92772 | 0.0040325 | 0.0138829 | 191 | 19120 |
| kmer: 15 -exp_cov 7 | 2728.35 | 379 | 7482 | 5419 | 3531 | 1430 | 0.933104 | 0.00528194 | 0.021701 | 317 | 19147 |
| kmer: 15 -exp_cov 13 | 2839.6 | 422 | 7515 | 5513 | 3553 | 1437 | 0.928376 | 0.00613774 | 0.0290426 | 444 | 19055 |
| kmer: 15 -exp_cov 15 | 2842.26 | 424 | 7515 | 5513 | 3557 | 1437 | 0.928471 | 0.00616019 | 0.0290645 | 444 | 19057 |
| kmer: 15 -exp_cov 17 | 2843.16 | 424 | 7515 | 5513 | 3557 | 1437 | 0.928518 | 0.00617715 | 0.0290814 | 444 | 19058 |
| kmer: 15 -exp_cov 19 | 2843.63 | 424 | 7515 | 5513 | 3557 | 1437 | 0.928566 | 0.00618453 | 0.0290881 | 444 | 19059 |
| kmer: 17 -exp_cov 5 | 1807.47 | 182 | 6306 | 4028 | 2435 | 839 | 0.940874 | 0.00352805 | 0.0106063 | 137 | 19351 |
| kmer: 17 -exp_cov 7 | 2443.27 | 304 | 6917 | 4805 | 3088 | 1238 | 0.943525 | 0.00430522 | 0.0159486 | 226 | 19359 |
| kmer: 19 -exp_cov 3 | 699.415 | 108 | 3357 | 1233 | 645 | 174 | 0.920507 | 0.00079603 | 0.0010019 | 3 | 18913 |
| kmer: 19 -exp_cov 5 | 1684.76 | 149 | 5832 | 3536 | 2134 | 781 | 0.945449 | 0.00331392 | 0.00972579 | 125 | 19448 |
| kmer: 19 -exp_cov 11 | 2045.39 | 224 | 6162 | 3904 | 2413 | 981 | 0.943914 | 0.00419285 | 0.0174666 | 256 | 19391 |
| kmer: 19 -exp_cov 15 | 2047.78 | 224 | 6166 | 3903 | 2416 | 980 | 0.943835 | 0.00422818 | 0.0176849 | 259 | 19390 |
| kmer: 19 -exp_cov 19 | 2048.25 | 224 | 6166 | 3903 | 2416 | 980 | 0.943889 | 0.00423199 | 0.0176887 | 259 | 19391 |
| kmer: 21 -exp_cov 9 | 1671.97 | 162 | 5405 | 3124 | 1927 | 757 | 0.945886 | 0.00373973 | 0.0143337 | 206 | 19453 |
| kmer: 21 -exp_cov 11 | 1674.73 | 164 | 5402 | 3118 | 1925 | 755 | 0.944869 | 0.00384582 | 0.0155348 | 227 | 19434 |
| kmer: 21 -exp_cov 13 | 1676.24 | 164 | 5400 | 3119 | 1925 | 754 | 0.944829 | 0.00386169 | 0.015613 | 228 | 19434 |
| kmer: 23 -exp_cov 5 | 1241.43 | 127 | 4495 | 2308 | 1411 | 493 | 0.947502 | 0.00283099 | 0.00737443 | 89 | 19524 |
| kmer: 23 -exp_cov 7 | 1315.99 | 134 | 4553 | 2372 | 1473 | 537 | 0.947998 | 0.00316813 | 0.00908427 | 115 | 19523 |
| kmer: 23 -exp_cov 13 | 1329.6 | 136 | 4562 | 2377 | 1477 | 533 | 0.945818 | 0.00345702 | 0.0116391 | 159 | 19484 |
| kmer: 23 -exp_cov 15 | 1329.77 | 136 | 4562 | 2377 | 1477 | 533 | 0.94588 | 0.00346121 | 0.0116433 | 159 | 19485 |
| kmer: 23 -exp_cov 19 | 1330.19 | 136 | 4563 | 2377 | 1477 | 533 | 0.945927 | 0.00347087 | 0.011653 | 159 | 19486 |
| kmer: 13 -exp_cov auto | 969.522 | 122 | 3485 | 1546 | 843 | 151 | 0.852562 | 0.00650431 | 0.0455667 | 722 | 17612 |
| kmer: 23 -exp_cov auto | 1147.32 | 140 | 4211 | 2012 | 1158 | 329 | 0.925807 | 0.00277424 | 0.0107875 | 153 | 19062 |
| kmer: 13 -exp_cov 3 | 254.867 | 101 | 936 | 240 | 132 | 9 | 0.780609 | 0.000526214 | 0.00104781 | 8 | 16166 |
| kmer: 13 -exp_cov 7 | 1974.95 | 195 | 6022 | 3643 | 2233 | 766 | 0.895958 | 0.00654854 | 0.0308403 | 467 | 18427 |
| kmer: 13 -exp_cov 11 | 2232.62 | 270 | 6245 | 3918 | 2395 | 865 | 0.896861 | 0.00731327 | 0.0361419 | 551 | 18408 |
| kmer: 13 -exp_cov 13 | 2238.58 | 271 | 6247 | 3936 | 2400 | 864 | 0.896906 | 0.00738583 | 0.0364864 | 556 | 18410 |
| kmer: 13 -exp_cov 17 | 2241.19 | 271 | 6247 | 3938 | 2402 | 864 | 0.896909 | 0.00741958 | 0.0367772 | 561 | 18410 |
| kmer: 15 -exp_cov 3 | 596.808 | 109 | 2648 | 913 | 502 | 144 | 0.903247 | 0.000607717 | 0.000800946 | 3 | 18595 |
| kmer: 15 -exp_cov 9 | 2812.79 | 409 | 7509 | 5489 | 3559 | 1460 | 0.930127 | 0.00572539 | 0.0262757 | 399 | 19088 |
| kmer: 15 -exp_cov 11 | 2833.32 | 421 | 7516 | 5514 | 3559 | 1440 | 0.928906 | 0.00605275 | 0.0282918 | 431 | 19066 |
| kmer: 17 -exp_cov 3 | 729.16 | 110 | 3423 | 1308 | 691 | 183 | 0.917313 | 0.000689378 | 0.000922136 | 4 | 18842 |
| kmer: 17 -exp_cov 9 | 2500.01 | 328 | 6945 | 4822 | 3108 | 1249 | 0.941051 | 0.00477098 | 0.0194231 | 284 | 19316 |
| kmer: 17 -exp_cov 11 | 2508.06 | 330 | 6933 | 4818 | 3094 | 1225 | 0.938054 | 0.00501897 | 0.0230458 | 348 | 19258 |
| kmer: 17 -exp_cov 13 | 2510.61 | 330 | 6933 | 4815 | 3085 | 1225 | 0.937851 | 0.00506328 | 0.0233695 | 353 | 19254 |
| kmer: 17 -exp_cov 15 | 2512.29 | 331 | 6934 | 4815 | 3087 | 1224 | 0.937785 | 0.00506541 | 0.0234603 | 355 | 19253 |
| kmer: 17 -exp_cov 17 | 2512.17 | 331 | 6933 | 4813 | 3086 | 1224 | 0.937875 | 0.00505987 | 0.0234547 | 355 | 19254 |
| kmer: 17 -exp_cov 19 | 2512.76 | 331 | 6933 | 4813 | 3086 | 1224 | 0.937878 | 0.00506152 | 0.0234564 | 355 | 19254 |
| kmer: 19 -exp_cov 7 | 2007.77 | 206 | 6150 | 3905 | 2409 | 980 | 0.947147 | 0.00389499 | 0.0128828 | 174 | 19460 |
| kmer: 19 -exp_cov 9 | 2040.06 | 221 | 6163 | 3914 | 2419 | 980 | 0.944573 | 0.00420417 | 0.0162519 | 234 | 19409 |
| kmer: 19 -exp_cov 13 | 2047.04 | 224 | 6166 | 3903 | 2416 | 981 | 0.943889 | 0.00421784 | 0.017606 | 258 | 19391 |
| kmer: 19 -exp_cov 17 | 2048.17 | 224 | 6166 | 3903 | 2416 | 980 | 0.94388 | 0.00423134 | 0.0176881 | 259 | 19391 |
| kmer: 21 -exp_cov 3 | 633.994 | 108 | 3068 | 1077 | 564 | 154 | 0.921076 | 0.000936791 | 0.00124573 | 5 | 18948 |
| kmer: 21 -exp_cov 5 | 1481.89 | 136 | 5230 | 2958 | 1802 | 661 | 0.94825 | 0.00311329 | 0.0082637 | 100 | 19520 |
| kmer: 21 -exp_cov 7 | 1654.75 | 158 | 5396 | 3124 | 1929 | 760 | 0.948203 | 0.00339208 | 0.0116115 | 161 | 19494 |
| kmer: 21 -exp_cov 15 | 1676.24 | 164 | 5399 | 3119 | 1925 | 754 | 0.944855 | 0.00386825 | 0.015626 | 228 | 19435 |
| kmer: 21 -exp_cov 17 | 1676.37 | 164 | 5399 | 3119 | 1925 | 754 | 0.94487 | 0.00387045 | 0.0156282 | 228 | 19435 |
| kmer: 21 -exp_cov 19 | 1676.49 | 164 | 5399 | 3119 | 1925 | 754 | 0.944888 | 0.00387389 | 0.0156316 | 228 | 19435 |
| kmer: 23 -exp_cov 3 | 561.892 | 106 | 2703 | 883 | 473 | 135 | 0.919774 | 0.00111541 | 0.00137958 | 4 | 18954 |
| kmer: 23 -exp_cov 9 | 1327.44 | 135 | 4563 | 2379 | 1479 | 536 | 0.946467 | 0.00341218 | 0.0108418 | 145 | 19497 |
| kmer: 23 -exp_cov 11 | 1328.95 | 136 | 4562 | 2377 | 1477 | 533 | 0.945923 | 0.00345251 | 0.0114455 | 155 | 19487 |
| kmer: 23 -exp_cov 17 | 1330.1 | 136 | 4563 | 2377 | 1477 | 533 | 0.945911 | 0.00346947 | 0.0116516 | 159 | 19486 |
| kmer: 15 -exp_cov auto | 2075.61 | 285 | 6178 | 3923 | 2342 | 824 | 0.911033 | 0.00554208 | 0.0299812 | 472 | 18722 |
| kmer: 17 -exp_cov auto | 2090.63 | 284 | 6325 | 4079 | 2470 | 897 | 0.93168 | 0.00444026 | 0.0189144 | 278 | 19143 |
| kmer: 19 -exp_cov auto | 1780.05 | 209 | 5745 | 3397 | 2045 | 742 | 0.936179 | 0.00370752 | 0.0147992 | 212 | 19241 |
| kmer: 21 -exp_cov auto | 1453.72 | 166 | 5013 | 2700 | 1605 | 525 | 0.934052 | 0.00316307 | 0.012242 | 175 | 19213 |

Table S1C - Evaluation of low sequencing depth assembly with EULER-SR.

| **Parameter Settings** | **Mean** | **Min** | **Max** | **N50** | **N75** | **N90** | **Coverage** | **Error** | **Total Error** | **Unmapped** | **All** |
| --- | --- | --- | --- | --- | --- | --- | --- | --- | --- | --- | --- |
| kmer:13 | 1405.71 | 164 | 4647 | 2357 | 1243 | 240 | 0.8473 | 0.018591 | 0.122636 | 2206 | 17558 |
| kmer:15 | 2035.51 | 235 | 6141 | 3779 | 2225 | 681 | 0.896943 | 0.018707 | 0.071304 | 1135 | 18524 |
| kmer:17 | 2291.79 | 303 | 6604 | 4339 | 2596 | 919 | 0.911728 | 0.020649 | 0.05336 | 724 | 18791 |
| kmer:19 | 2374.22 | 339 | 6684 | 4484 | 2732 | 900 | 0.912173 | 0.023134 | 0.051188 | 584 | 18790 |
| kmer:21 | 2374.27 | 349 | 6645 | 4452 | 2704 | 828 | 0.907206 | 0.026845 | 0.053012 | 520 | 18684 |
| kmer:23 | 2295.26 | 303 | 6522 | 4221 | 2480 | 628 | 0.892354 | 0.03035 | 0.062604 | 639 | 18388 |

Table S1D - Evaluation of low sequencing depth assembly with ABySS.

| **Parameter Settings** | **Mean** | **Min** | **Max** | **N50** | **N75** | **N90** | **Coverage** | **Error** | **Total Error** | **Unmapped** | **All** |
| --- | --- | --- | --- | --- | --- | --- | --- | --- | --- | --- | --- |
| kmer:13 n=2 b=26 c=1.4 e=0 | 618.633 | 106 | 2400 | 848 | 433 | 48 | 0.807374 | 0.00132551 | 0.00773125 | 118 | 17348 |
| kmer:13 n=2 b=26 c=1.4 e=1 | 626.353 | 107 | 2381 | 822 | 406 | 30 | 0.791088 | 0.00141369 | 0.00990267 | 155 | 16951 |
| kmer:13 n=2 b=52 c=1.2 e=0 | 661.052 | 106 | 2525 | 937 | 492 | 64 | 0.819076 | 0.00157451 | 0.00662996 | 93 | 17658 |
| kmer:13 n=2 b=52 c=2.0 e=1 | 522.453 | 105 | 2006 | 636 | 288 | 7 | 0.770684 | 0.00169404 | 0.0118594 | 176 | 16373 |
| kmer:13 n=6 b=26 c=1.4 e=0 | 301.688 | 101 | 1255 | 322 | 148 | 8 | 0.747779 | 0.000499733 | 0.00288505 | 37 | 15528 |
| kmer:13 n=6 b=26 c=2.0 e=1 | 282.768 | 101 | 1140 | 268 | 108 | 0 | 0.713912 | 0.000483216 | 0.00435704 | 57 | 14811 |
| kmer:13 n=6 b=52 c=1.0 e=0 | 107.949 | 68 | 276 | 8 | 0 | 0 | 0.274155 | 0.000346943 | 0.000446663 | 0 | 5643 |
| kmer:13 n=6 b=52 c=1.4 e=1 | 306.087 | 101 | 1257 | 318 | 140 | 4 | 0.735762 | 0.000601071 | 0.0034066 | 43 | 15274 |
| kmer:13 n=10 b=26 c=2.0 e=0 | 225.416 | 100 | 813 | 187 | 84 | 0 | 0.714339 | 0.000320423 | 0.00248768 | 32 | 14880 |
| kmer:13 n=10 b=52 c=1.0 e=0 | 107.942 | 68 | 276 | 8 | 0 | 0 | 0.274152 | 0.000346945 | 0.000446664 | 0 | 5643 |
| kmer:13 n=10 b=52 c=1.0 e=1 | 113.846 | 71 | 291 | 8 | 0 | 0 | 0.285315 | 0.000297509 | 0.000440918 | 1 | 5871 |
| kmer:13 n=10 b=52 c=1.4 e=0 | 232.351 | 100 | 858 | 204 | 99 | 1 | 0.735133 | 0.00042698 | 0.00156194 | 17 | 15327 |
| kmer:13 n=10 b=52 c=2.0 e=0 | 225.721 | 101 | 815 | 189 | 86 | 0 | 0.71686 | 0.000392867 | 0.00259216 | 32 | 14932 |
| kmer:15 n=2 b=30 c=1.0 e=0 | 156.061 | 91 | 431 | 37 | 0 | 0 | 0.430557 | 0.000592753 | 0.000902728 | 3 | 8833 |
| kmer:15 n=2 b=60 c=1.2 e=0 | 1285.01 | 139 | 4391 | 2228 | 1343 | 419 | 0.922521 | 0.00154413 | 0.00643759 | 100 | 19309 |
| kmer:15 n=2 b=60 c=2.0 e=1 | 774.969 | 117 | 2811 | 1107 | 615 | 77 | 0.867652 | 0.00165657 | 0.0104275 | 162 | 18016 |
| kmer:15 n=6 b=30 c=1.0 e=0 | 153.455 | 91 | 406 | 35 | 0 | 0 | 0.428393 | 0.000552913 | 0.000812961 | 2 | 8787 |
| kmer:15 n=6 b=30 c=1.2 e=1 | 1083.94 | 137 | 3817 | 1717 | 955 | 191 | 0.875401 | 0.0008835 | 0.00444816 | 65 | 18038 |
| kmer:15 n=6 b=30 c=1.4 e=1 | 974.966 | 127 | 3499 | 1508 | 835 | 157 | 0.872142 | 0.000886096 | 0.00444411 | 64 | 17946 |
| kmer:15 n=6 b=30 c=2.0 e=1 | 704.642 | 112 | 2677 | 1009 | 539 | 64 | 0.854341 | 0.000913508 | 0.00579969 | 85 | 17566 |
| kmer:15 n=6 b=60 c=1.4 e=1 | 996.531 | 129 | 3539 | 1543 | 863 | 165 | 0.877332 | 0.0012555 | 0.0057383 | 82 | 18078 |
| kmer:15 n=10 b=60 c=2.0 e=1 | 627.469 | 109 | 2503 | 902 | 475 | 54 | 0.852761 | 0.000897237 | 0.00507287 | 72 | 17525 |
| kmer:17 n=2 b=68 c=1.2 e=0 | 1472.93 | 154 | 4786 | 2577 | 1545 | 486 | 0.924247 | 0.00149577 | 0.0070371 | 113 | 19188 |
| kmer:17 n=2 b=68 c=1.2 e=1 | 1420 | 182 | 4386 | 2189 | 1268 | 272 | 0.885655 | 0.00147603 | 0.012182 | 215 | 18356 |
| kmer:17 n=6 b=34 c=1.4 e=0 | 1138.85 | 132 | 4053 | 1933 | 1126 | 302 | 0.908508 | 0.000881995 | 0.00303901 | 41 | 18678 |
| kmer:17 n=6 b=68 c=1.4 e=0 | 1172.41 | 134 | 4111 | 1986 | 1167 | 321 | 0.913599 | 0.00131358 | 0.00478942 | 67 | 18826 |
| kmer:17 n=6 b=68 c=2.0 e=1 | 760.735 | 118 | 2745 | 1069 | 584 | 73 | 0.861261 | 0.0012687 | 0.00576477 | 79 | 17725 |
| kmer:17 n=10 b=34 c=2.0 e=0 | 707.062 | 113 | 2758 | 1065 | 597 | 115 | 0.885226 | 0.000707599 | 0.00255526 | 33 | 18180 |
| kmer:17 n=10 b=34 c=2.0 e=1 | 696.879 | 114 | 2642 | 984 | 517 | 61 | 0.852651 | 0.000628796 | 0.003117 | 43 | 17496 |
| kmer:17 n=10 b=68 c=1.0 e=1 | 293.545 | 103 | 1036 | 252 | 102 | 0 | 0.721835 | 0.000465136 | 0.000730331 | 3 | 14790 |
| kmer:17 n=10 b=68 c=2.0 e=0 | 717.567 | 114 | 2775 | 1082 | 611 | 118 | 0.8887 | 0.000966802 | 0.0039614 | 54 | 18261 |
| kmer:19 n=2 b=38 c=1.4 e=0 | 1234.24 | 138 | 4202 | 2068 | 1204 | 318 | 0.908453 | 0.00121349 | 0.0045018 | 64 | 18805 |
| kmer:19 n=6 b=38 c=1.4 e=1 | 1103.51 | 141 | 3732 | 1691 | 949 | 188 | 0.877394 | 0.000815607 | 0.00306824 | 42 | 18009 |
| kmer:19 n=6 b=76 c=1.4 e=0 | 1195.76 | 133 | 4245 | 2124 | 1299 | 483 | 0.950731 | 0.00127938 | 0.00279693 | 30 | 19602 |
| kmer:19 n=6 b=76 c=2.0 e=0 | 752.814 | 116 | 2835 | 1172 | 707 | 200 | 0.925958 | 0.00117703 | 0.00295503 | 33 | 19069 |
| kmer:19 n=10 b=38 c=1.0 e=0 | 173.683 | 99 | 479 | 97 | 0 | 0 | 0.541871 | 0.00079925 | 0.000816189 | 0 | 11155 |
| kmer:19 n=10 b=38 c=2.0 e=0 | 691.614 | 113 | 2718 | 1083 | 638 | 175 | 0.918133 | 0.000704966 | 0.00175166 | 20 | 18859 |
| kmer:13 n=10 b=26 c=2.0 e=1 | 225.961 | 101 | 809 | 183 | 77 | 0 | 0.700283 | 0.00032107 | 0.00281744 | 36 | 14584 |
| kmer:13 n=10 b=26 c=1.2 e=1 | 234.455 | 101 | 862 | 201 | 93 | 0 | 0.721023 | 0.000333209 | 0.00147942 | 17 | 15032 |
| kmer:13 n=6 b=52 c=1.0 e=1 | 113.857 | 71 | 291 | 8 | 0 | 0 | 0.285318 | 0.000297508 | 0.000440916 | 1 | 5872 |
| kmer:13 n=10 b=52 c=1.4 e=1 | 233.096 | 101 | 856 | 199 | 91 | 0 | 0.719778 | 0.000397486 | 0.00183067 | 21 | 15002 |
| kmer:13 n=10 b=26 c=1.0 e=0 | 31.0096 | 21 | 74 | 0 | 0 | 0 | 0.0572849 | 0.000122125 | 0.00014746 | 0 | 1175 |
| kmer:15 n=2 b=30 c=1.2 e=0 | 1251.79 | 134 | 4334 | 2180 | 1289 | 397 | 0.917431 | 0.00110599 | 0.00556166 | 90 | 19232 |
| kmer:13 n=10 b=52 c=1.2 e=1 | 235.088 | 101 | 868 | 203 | 95 | 0 | 0.724311 | 0.000404167 | 0.00156518 | 17 | 15101 |
| kmer:13 n=2 b=26 c=1.0 e=1 | 33.3345 | 22 | 79 | 0 | 0 | 0 | 0.060689 | 0.000108163 | 0.000174454 | 0 | 1245 |
| kmer:15 n=2 b=30 c=2.0 e=1 | 763.783 | 116 | 2792 | 1088 | 599 | 74 | 0.863059 | 0.00117025 | 0.00937568 | 151 | 17892 |
| kmer:15 n=2 b=60 c=1.0 e=0 | 317.506 | 101 | 1260 | 293 | 100 | 0 | 0.717079 | 0.000889377 | 0.00183652 | 13 | 15084 |
| kmer:15 n=6 b=30 c=1.2 e=0 | 1086.7 | 125 | 4014 | 1910 | 1106 | 315 | 0.906071 | 0.000934629 | 0.00316592 | 41 | 18676 |
| kmer:15 n=6 b=30 c=2.0 e=0 | 713.21 | 112 | 2774 | 1080 | 602 | 120 | 0.881589 | 0.000932658 | 0.00484998 | 70 | 18132 |
| kmer:15 n=6 b=60 c=1.0 e=1 | 246.433 | 101 | 844 | 187 | 60 | 0 | 0.677952 | 0.000424978 | 0.00100532 | 7 | 13916 |
| kmer:15 n=10 b=30 c=1.2 e=0 | 890.399 | 114 | 3541 | 1551 | 878 | 235 | 0.899675 | 0.000784739 | 0.00221735 | 26 | 18503 |
| kmer:15 n=10 b=60 c=1.0 e=0 | 230.412 | 101 | 765 | 178 | 66 | 0 | 0.686389 | 0.000575268 | 0.000864052 | 4 | 14125 |
| kmer:17 n=2 b=34 c=1.0 e=0 | 179.534 | 97 | 544 | 82 | 0 | 0 | 0.513658 | 0.000738313 | 0.00103859 | 3 | 10548 |
| kmer:17 n=2 b=68 c=1.0 e=1 | 441.934 | 104 | 1756 | 509 | 176 | 0 | 0.758317 | 0.000753954 | 0.00176447 | 15 | 16104 |
| kmer:17 n=2 b=68 c=2.0 e=1 | 794.44 | 120 | 2812 | 1129 | 620 | 78 | 0.865787 | 0.00146747 | 0.00867351 | 133 | 17869 |
| kmer:17 n=6 b=34 c=1.2 e=1 | 1245.42 | 157 | 4094 | 1951 | 1099 | 216 | 0.875391 | 0.000756864 | 0.00434511 | 66 | 17983 |
| kmer:17 n=6 b=68 c=1.4 e=1 | 1131.9 | 144 | 3788 | 1741 | 979 | 178 | 0.877439 | 0.00117106 | 0.00635387 | 97 | 18061 |
| kmer:17 n=10 b=68 c=1.4 e=1 | 1030.29 | 134 | 3637 | 1616 | 886 | 151 | 0.872781 | 0.000877649 | 0.00447204 | 65 | 17922 |
| kmer:19 n=2 b=38 c=1.4 e=1 | 1173.54 | 147 | 3837 | 1758 | 963 | 167 | 0.866869 | 0.00114381 | 0.00646855 | 100 | 17897 |
| kmer:19 n=2 b=76 c=1.0 e=1 | 506.333 | 105 | 1951 | 617 | 228 | 0 | 0.772032 | 0.000750917 | 0.00171212 | 15 | 16432 |
| kmer:19 n=6 b=38 c=1.0 e=1 | 190.985 | 100 | 552 | 101 | 0 | 0 | 0.551583 | 0.000487211 | 0.000538634 | 0 | 11288 |
| kmer:19 n=6 b=38 c=1.2 e=1 | 1267.52 | 155 | 4140 | 1995 | 1127 | 244 | 0.88089 | 0.000790807 | 0.00280971 | 38 | 18091 |
| kmer:19 n=6 b=76 c=1.2 e=0 | 1419.95 | 143 | 4882 | 2677 | 1627 | 647 | 0.956181 | 0.00134926 | 0.00280601 | 31 | 19733 |
| kmer:19 n=6 b=76 c=2.0 e=1 | 730.407 | 117 | 2693 | 1067 | 621 | 106 | 0.893034 | 0.0010733 | 0.00424626 | 60 | 18369 |
| kmer:19 n=10 b=38 c=1.2 e=1 | 1162.18 | 144 | 4007 | 1919 | 1130 | 298 | 0.90836 | 0.000600674 | 0.00122535 | 11 | 18625 |
| kmer:19 n=10 b=38 c=2.0 e=1 | 681.257 | 113 | 2612 | 998 | 565 | 93 | 0.885518 | 0.00056256 | 0.00202335 | 27 | 18157 |
| kmer:19 n=10 b=76 c=1.2 e=0 | 1250.49 | 129 | 4579 | 2380 | 1436 | 534 | 0.95084 | 0.00110827 | 0.00344969 | 49 | 19561 |
| kmer:19 n=10 b=76 c=1.2 e=1 | 1233.82 | 149 | 4199 | 2057 | 1220 | 328 | 0.912934 | 0.000865545 | 0.00271929 | 37 | 18740 |
| kmer:13 n=6 b=26 c=1.2 e=0 | 306.993 | 101 | 1280 | 335 | 155 | 11 | 0.752554 | 0.000524783 | 0.00248523 | 31 | 15632 |
| kmer:15 n=6 b=60 c=1.4 e=0 | 1006.85 | 124 | 3723 | 1691 | 979 | 273 | 0.907474 | 0.00128356 | 0.00435885 | 58 | 18714 |
| kmer:13 n=2 b=26 c=1.0 e=0 | 31.033 | 21 | 74 | 0 | 0 | 0 | 0.0573087 | 0.000122269 | 0.000147604 | 0 | 1176 |
| kmer:15 n=2 b=30 c=1.2 e=1 | 1236.72 | 150 | 4083 | 1947 | 1110 | 239 | 0.885055 | 0.00118906 | 0.00996046 | 175 | 18514 |
| kmer:15 n=2 b=60 c=1.4 e=0 | 1130.47 | 134 | 3947 | 1872 | 1112 | 329 | 0.917482 | 0.00148301 | 0.00674362 | 105 | 19169 |
| kmer:13 n=6 b=26 c=1.4 e=1 | 304.702 | 101 | 1252 | 314 | 138 | 4 | 0.732585 | 0.000489172 | 0.00319495 | 42 | 15207 |
| kmer:15 n=2 b=30 c=2.0 e=0 | 775.915 | 115 | 2901 | 1171 | 672 | 137 | 0.890941 | 0.00110183 | 0.00711426 | 114 | 18488 |
| kmer:15 n=2 b=60 c=1.0 e=1 | 339.434 | 102 | 1351 | 314 | 96 | 0 | 0.708527 | 0.000768475 | 0.0022784 | 22 | 14930 |
| kmer:15 n=6 b=30 c=1.0 e=1 | 160.531 | 94 | 426 | 30 | 0 | 0 | 0.434516 | 0.000387457 | 0.000759749 | 3 | 8905 |
| kmer:13 n=2 b=52 c=1.4 e=1 | 630.795 | 107 | 2391 | 832 | 416 | 30 | 0.795344 | 0.00165013 | 0.0101471 | 156 | 17051 |
| kmer:13 n=10 b=26 c=1.2 e=0 | 233.487 | 100 | 868 | 205 | 101 | 2 | 0.736607 | 0.000370546 | 0.00120682 | 12 | 15362 |
| kmer:13 n=2 b=52 c=1.4 e=0 | 623.1 | 106 | 2408 | 860 | 443 | 49 | 0.81168 | 0.00155459 | 0.00790557 | 118 | 17451 |
| kmer:15 n=6 b=60 c=2.0 e=0 | 724.467 | 112 | 2795 | 1096 | 619 | 125 | 0.886818 | 0.00131708 | 0.00590636 | 84 | 18258 |
| kmer:17 n=2 b=34 c=1.0 e=1 | 188.625 | 99 | 587 | 78 | 0 | 0 | 0.514437 | 0.000535101 | 0.000975073 | 4 | 10552 |
| kmer:17 n=2 b=68 c=1.4 e=1 | 1214.14 | 154 | 3902 | 1850 | 1058 | 202 | 0.882703 | 0.00147195 | 0.0105776 | 179 | 18278 |
| kmer:17 n=2 b=68 c=2.0 e=0 | 817.62 | 120 | 2968 | 1228 | 716 | 149 | 0.898974 | 0.00144594 | 0.00662885 | 98 | 18571 |
| kmer:17 n=6 b=34 c=2.0 e=1 | 743.929 | 117 | 2718 | 1047 | 563 | 69 | 0.856497 | 0.000842455 | 0.00409443 | 57 | 17592 |
| kmer:17 n=10 b=34 c=1.2 e=1 | 1121.54 | 145 | 3883 | 1779 | 982 | 185 | 0.871741 | 0.00060924 | 0.00279041 | 39 | 17883 |
| kmer:17 n=10 b=34 c=1.4 e=1 | 1006.2 | 132 | 3586 | 1569 | 857 | 144 | 0.868568 | 0.000592404 | 0.00323248 | 48 | 17819 |
| kmer:17 n=10 b=68 c=2.0 e=1 | 707.598 | 115 | 2659 | 1001 | 532 | 62 | 0.856249 | 0.000882067 | 0.00451206 | 63 | 17577 |
| kmer:19 n=2 b=38 c=1.2 e=0 | 1445.91 | 147 | 4759 | 2531 | 1494 | 428 | 0.912634 | 0.00122978 | 0.00531515 | 81 | 19003 |
| kmer:19 n=2 b=38 c=2.0 e=0 | 777.393 | 119 | 2843 | 1142 | 649 | 122 | 0.885835 | 0.0011555 | 0.004885 | 69 | 18268 |
| kmer:19 n=2 b=38 c=2.0 e=1 | 749.558 | 118 | 2679 | 1024 | 541 | 58 | 0.848288 | 0.00111395 | 0.00610242 | 89 | 17461 |
| kmer:19 n=2 b=76 c=1.4 e=0 | 1261.63 | 140 | 4258 | 2139 | 1285 | 404 | 0.926861 | 0.00148333 | 0.00445885 | 60 | 19200 |
| kmer:19 n=6 b=38 c=1.2 e=0 | 1324.3 | 138 | 4576 | 2378 | 1404 | 445 | 0.92205 | 0.000989264 | 0.00216632 | 22 | 18978 |
| kmer:19 n=6 b=38 c=1.4 e=0 | 1153.74 | 132 | 4094 | 1978 | 1164 | 343 | 0.917808 | 0.000973102 | 0.002191 | 23 | 18873 |
| kmer:19 n=6 b=38 c=2.0 e=0 | 740.985 | 116 | 2812 | 1145 | 676 | 181 | 0.91642 | 0.000848013 | 0.0018599 | 18 | 18834 |
| kmer:19 n=10 b=76 c=1.0 e=1 | 323.893 | 103 | 1157 | 312 | 155 | 0 | 0.770038 | 0.000518042 | 0.00061476 | 1 | 15768 |
| kmer:19 n=10 b=76 c=1.4 e=0 | 1074.02 | 126 | 4020 | 1921 | 1162 | 413 | 0.945581 | 0.00105202 | 0.00296293 | 38 | 19452 |
| kmer:19 n=10 b=76 c=1.4 e=1 | 1050.32 | 135 | 3701 | 1696 | 993 | 245 | 0.908409 | 0.000834801 | 0.00314566 | 46 | 18641 |
| kmer:13 n=10 b=26 c=1.4 e=1 | 232.841 | 101 | 854 | 198 | 90 | 0 | 0.717028 | 0.000319341 | 0.00174675 | 21 | 14945 |
| kmer:13 n=10 b=26 c=1.4 e=0 | 232.085 | 100 | 856 | 202 | 98 | 1 | 0.732418 | 0.000350529 | 0.00147822 | 17 | 15271 |
| kmer:17 n=6 b=34 c=1.0 e=0 | 172.95 | 97 | 477 | 78 | 0 | 0 | 0.508416 | 0.000660917 | 0.000791307 | 1 | 10428 |
| kmer:13 n=2 b=26 c=2.0 e=0 | 515.827 | 104 | 2023 | 649 | 301 | 16 | 0.781526 | 0.00135664 | 0.00959922 | 143 | 16622 |
| kmer:17 n=2 b=68 c=1.0 e=0 | 403.068 | 103 | 1632 | 467 | 175 | 0 | 0.772055 | 0.000931405 | 0.00147128 | 8 | 16371 |
| kmer:15 n=2 b=60 c=1.4 e=1 | 1110.28 | 140 | 3723 | 1690 | 971 | 198 | 0.886176 | 0.00159586 | 0.0107016 | 179 | 18485 |
| kmer:13 n=2 b=52 c=2.0 e=0 | 518.401 | 104 | 2027 | 655 | 306 | 16 | 0.785341 | 0.00158639 | 0.00988469 | 145 | 16703 |
| kmer:15 n=6 b=60 c=2.0 e=1 | 715.98 | 113 | 2698 | 1025 | 556 | 67 | 0.85952 | 0.00130808 | 0.00686898 | 99 | 17689 |
| kmer:17 n=2 b=34 c=1.2 e=1 | 1377.76 | 173 | 4309 | 2139 | 1227 | 258 | 0.88235 | 0.000995425 | 0.00932602 | 165 | 18349 |
| kmer:17 n=6 b=68 c=1.2 e=0 | 1348.92 | 142 | 4599 | 2396 | 1413 | 421 | 0.917928 | 0.00132769 | 0.0044961 | 62 | 18921 |
| kmer:15 n=10 b=60 c=1.2 e=1 | 933.996 | 122 | 3537 | 1516 | 831 | 153 | 0.874223 | 0.00094632 | 0.00356391 | 47 | 17971 |
| kmer:13 n=6 b=26 c=1.0 e=1 | 33.3015 | 22 | 78 | 0 | 0 | 0 | 0.0606537 | 0.000107906 | 0.000174197 | 0 | 1244 |
| kmer:15 n=10 b=60 c=1.2 e=0 | 923.556 | 115 | 3662 | 1639 | 931 | 251 | 0.904495 | 0.00100949 | 0.00276317 | 32 | 18610 |
| kmer:13 n=6 b=52 c=1.4 e=0 | 303.031 | 101 | 1260 | 326 | 150 | 8 | 0.750921 | 0.000611136 | 0.00306061 | 39 | 15595 |
| kmer:15 n=10 b=60 c=2.0 e=0 | 629.999 | 109 | 2578 | 955 | 524 | 101 | 0.879813 | 0.000938488 | 0.00428565 | 60 | 18093 |
| kmer:15 n=10 b=60 c=1.4 e=1 | 839.435 | 116 | 3220 | 1321 | 722 | 122 | 0.870586 | 0.000922195 | 0.00369286 | 49 | 17892 |
| kmer:17 n=2 b=68 c=1.4 e=0 | 1266.9 | 143 | 4268 | 2116 | 1272 | 365 | 0.92022 | 0.00150171 | 0.00618145 | 92 | 19079 |
| kmer:15 n=10 b=60 c=1.4 e=0 | 837.432 | 113 | 3351 | 1420 | 805 | 205 | 0.900123 | 0.000983743 | 0.00292983 | 35 | 18513 |
| kmer:13 n=10 b=52 c=1.2 e=0 | 234.128 | 100 | 874 | 207 | 103 | 2 | 0.73987 | 0.000441465 | 0.00128576 | 13 | 15430 |
| kmer:17 n=2 b=34 c=1.4 e=0 | 1240.66 | 140 | 4241 | 2082 | 1239 | 350 | 0.916166 | 0.0010389 | 0.00505069 | 78 | 18956 |
| kmer:17 n=6 b=34 c=1.2 e=0 | 1282.26 | 137 | 4446 | 2261 | 1332 | 386 | 0.912171 | 0.000919744 | 0.00302348 | 40 | 18764 |
| kmer:13 n=10 b=26 c=1.0 e=1 | 33.3015 | 22 | 78 | 0 | 0 | 0 | 0.0606537 | 0.000107906 | 0.000174197 | 0 | 1244 |
| kmer:15 n=2 b=30 c=1.0 e=1 | 163.627 | 94 | 457 | 32 | 0 | 0 | 0.437017 | 0.000436834 | 0.000914015 | 4 | 8958 |
| kmer:13 n=6 b=26 c=1.0 e=0 | 31.0096 | 21 | 74 | 0 | 0 | 0 | 0.0572849 | 0.000122125 | 0.00014746 | 0 | 1175 |
| kmer:13 n=6 b=26 c=1.2 e=1 | 310.642 | 101 | 1278 | 329 | 145 | 5 | 0.737136 | 0.00050118 | 0.00279614 | 36 | 15307 |
| kmer:17 n=6 b=34 c=1.0 e=1 | 180.618 | 99 | 504 | 74 | 0 | 0 | 0.508253 | 0.000436061 | 0.000626191 | 1 | 10404 |
| kmer:13 n=6 b=52 c=1.2 e=0 | 309.988 | 101 | 1294 | 342 | 159 | 12 | 0.756395 | 0.000632988 | 0.00259464 | 31 | 15711 |
| kmer:17 n=6 b=68 c=2.0 e=0 | 779.122 | 117 | 2895 | 1170 | 674 | 136 | 0.893908 | 0.00129029 | 0.00500331 | 68 | 18409 |
| kmer:17 n=10 b=34 c=1.4 e=0 | 1024.06 | 125 | 3833 | 1762 | 1011 | 256 | 0.904574 | 0.000735406 | 0.0020309 | 24 | 18579 |
| kmer:17 n=10 b=68 c=1.0 e=0 | 268.282 | 102 | 933 | 233 | 109 | 0 | 0.734926 | 0.000667116 | 0.000857826 | 2 | 15116 |
| kmer:17 n=10 b=68 c=1.2 e=0 | 1187.91 | 128 | 4325 | 2133 | 1242 | 347 | 0.912456 | 0.00106223 | 0.00359055 | 49 | 18761 |
| kmer:17 n=10 b=68 c=1.2 e=1 | 1180.21 | 148 | 4040 | 1889 | 1043 | 201 | 0.876065 | 0.000923884 | 0.00453141 | 68 | 17995 |
| kmer:17 n=10 b=68 c=1.4 e=0 | 1047.9 | 125 | 3889 | 1813 | 1043 | 267 | 0.908694 | 0.00102404 | 0.00334705 | 43 | 18680 |
| kmer:19 n=2 b=76 c=1.0 e=0 | 453.22 | 103 | 1793 | 562 | 217 | 1 | 0.786904 | 0.000995465 | 0.00163498 | 10 | 16743 |
| kmer:19 n=2 b=76 c=1.2 e=0 | 1501.68 | 153 | 4908 | 2700 | 1619 | 550 | 0.931175 | 0.00151096 | 0.00573998 | 87 | 19294 |
| kmer:19 n=2 b=76 c=2.0 e=0 | 786.123 | 119 | 2863 | 1180 | 689 | 158 | 0.903152 | 0.00137406 | 0.00436742 | 57 | 18658 |
| kmer:19 n=2 b=76 c=2.0 e=1 | 758.31 | 119 | 2700 | 1064 | 585 | 79 | 0.86672 | 0.00132708 | 0.00563362 | 80 | 17869 |
| kmer:19 n=6 b=76 c=1.2 e=1 | 1341.7 | 161 | 4356 | 2207 | 1327 | 380 | 0.917528 | 0.00109446 | 0.00349797 | 49 | 18884 |
| kmer:19 n=10 b=38 c=1.0 e=1 | 190.973 | 100 | 553 | 112 | 0 | 0 | 0.56711 | 0.000480332 | 0.000529782 | 0 | 11607 |
| kmer:19 n=10 b=38 c=1.4 e=1 | 1025.61 | 133 | 3654 | 1656 | 964 | 234 | 0.904685 | 0.000595072 | 0.00160937 | 20 | 18553 |
| kmer:19 n=10 b=76 c=2.0 e=0 | 700.65 | 113 | 2736 | 1099 | 649 | 179 | 0.921022 | 0.000921789 | 0.00288952 | 37 | 18936 |
| kmer:19 n=10 b=76 c=2.0 e=1 | 690.445 | 114 | 2628 | 1012 | 578 | 95 | 0.888545 | 0.000785791 | 0.00339023 | 48 | 18235 |
| kmer:15 n=6 b=60 c=1.0 e=0 | 233.877 | 101 | 788 | 181 | 67 | 0 | 0.686904 | 0.000584658 | 0.000941271 | 4 | 14127 |
| kmer:17 n=6 b=34 c=2.0 e=0 | 762.822 | 116 | 2871 | 1147 | 653 | 129 | 0.889303 | 0.000889293 | 0.0032079 | 42 | 18277 |
| kmer:13 n=6 b=52 c=2.0 e=1 | 283.912 | 101 | 1144 | 271 | 110 | 0 | 0.716867 | 0.000598951 | 0.0045545 | 59 | 14874 |
| kmer:13 n=10 b=52 c=2.0 e=1 | 226.27 | 101 | 812 | 184 | 78 | 0 | 0.70283 | 0.000396717 | 0.00292257 | 37 | 14636 |
| kmer:17 n=2 b=34 c=2.0 e=1 | 781.822 | 119 | 2792 | 1109 | 603 | 76 | 0.862173 | 0.000989861 | 0.00738118 | 118 | 17753 |
| kmer:19 n=2 b=76 c=1.2 e=1 | 1425.73 | 176 | 4422 | 2233 | 1290 | 302 | 0.889559 | 0.00141873 | 0.00763134 | 122 | 18369 |
| kmer:15 n=10 b=30 c=2.0 e=0 | 624.631 | 108 | 2561 | 944 | 513 | 98 | 0.875984 | 0.000726106 | 0.00341932 | 48 | 18009 |
| kmer:17 n=2 b=34 c=1.4 e=1 | 1188.84 | 150 | 3876 | 1819 | 1028 | 193 | 0.878719 | 0.00099465 | 0.00912932 | 158 | 18156 |
| kmer:15 n=6 b=60 c=1.2 e=1 | 1128.45 | 140 | 3907 | 1795 | 1015 | 207 | 0.881097 | 0.00127163 | 0.00557384 | 79 | 18174 |
| kmer:19 n=10 b=38 c=1.2 e=0 | 1181.09 | 128 | 4361 | 2200 | 1329 | 489 | 0.946647 | 0.000899715 | 0.00158872 | 13 | 19463 |
| kmer:17 n=10 b=34 c=1.0 e=1 | 180.607 | 99 | 504 | 74 | 0 | 0 | 0.508252 | 0.000436062 | 0.000626193 | 1 | 10404 |
| kmer:19 n=2 b=38 c=1.0 e=1 | 204.752 | 100 | 686 | 103 | 0 | 0 | 0.552518 | 0.000626602 | 0.000972795 | 4 | 11340 |
| kmer:15 n=10 b=60 c=1.0 e=1 | 242.526 | 101 | 820 | 184 | 60 | 0 | 0.677365 | 0.000411687 | 0.000949459 | 7 | 13914 |
| kmer:17 n=2 b=34 c=1.2 e=0 | 1427.94 | 149 | 4708 | 2510 | 1497 | 461 | 0.920443 | 0.00103995 | 0.00515405 | 83 | 19171 |
| kmer:13 n=2 b=26 c=1.2 e=1 | 664.498 | 109 | 2481 | 892 | 453 | 39 | 0.797742 | 0.00145859 | 0.0087474 | 134 | 17156 |
| kmer:15 n=10 b=30 c=1.0 e=0 | 153.449 | 91 | 406 | 35 | 0 | 0 | 0.428392 | 0.000552913 | 0.000812961 | 2 | 8787 |
| kmer:13 n=6 b=26 c=2.0 e=0 | 280.668 | 101 | 1140 | 273 | 118 | 2 | 0.727856 | 0.000475321 | 0.00385978 | 51 | 15104 |
| kmer:19 n=2 b=76 c=1.4 e=1 | 1196.86 | 151 | 3867 | 1815 | 1038 | 224 | 0.886025 | 0.00141474 | 0.00650609 | 99 | 18300 |
| kmer:13 n=2 b=52 c=1.0 e=1 | 116.209 | 71 | 308 | 9 | 0 | 0 | 0.287016 | 0.000319241 | 0.000573965 | 2 | 5909 |
| kmer:13 n=2 b=26 c=1.2 e=0 | 653.154 | 106 | 2507 | 920 | 480 | 61 | 0.814148 | 0.00134764 | 0.0063893 | 93 | 17552 |
| kmer:13 n=6 b=52 c=2.0 e=0 | 281.766 | 101 | 1144 | 276 | 119 | 2 | 0.730775 | 0.000591976 | 0.00403636 | 52 | 15166 |
| kmer:19 n=6 b=38 c=2.0 e=1 | 717.202 | 116 | 2671 | 1051 | 603 | 101 | 0.889298 | 0.000744529 | 0.00226999 | 28 | 18253 |
| kmer:19 n=2 b=38 c=1.0 e=0 | 193.508 | 99 | 626 | 105 | 0 | 0 | 0.555843 | 0.000881757 | 0.00106826 | 2 | 11431 |
| kmer:15 n=10 b=30 c=1.0 e=1 | 160.527 | 94 | 426 | 30 | 0 | 0 | 0.434513 | 0.000387458 | 0.000759749 | 3 | 8905 |
| kmer:17 n=10 b=34 c=1.2 e=0 | 1129.78 | 127 | 4152 | 1992 | 1160 | 320 | 0.907846 | 0.000768709 | 0.00224056 | 27 | 18647 |
| kmer:19 n=6 b=76 c=1.0 e=1 | 331.402 | 103 | 1200 | 324 | 159 | 0 | 0.771742 | 0.000544322 | 0.00068076 | 2 | 15799 |
| kmer:13 n=6 b=52 c=1.2 e=1 | 313.537 | 101 | 1291 | 335 | 149 | 5 | 0.740965 | 0.000604436 | 0.00295846 | 37 | 15386 |
| kmer:15 n=6 b=30 c=1.4 e=0 | 985.68 | 122 | 3680 | 1656 | 951 | 261 | 0.902134 | 0.000921569 | 0.00314725 | 41 | 18581 |
| kmer:15 n=10 b=30 c=1.4 e=0 | 824.753 | 113 | 3309 | 1389 | 783 | 198 | 0.895721 | 0.000765283 | 0.00231866 | 28 | 18416 |
| kmer:15 n=6 b=60 c=1.2 e=0 | 1129.11 | 128 | 4109 | 2002 | 1175 | 338 | 0.911786 | 0.0012986 | 0.00430535 | 57 | 18812 |
| kmer:15 n=2 b=60 c=2.0 e=0 | 786.841 | 116 | 2919 | 1190 | 689 | 142 | 0.895652 | 0.00155268 | 0.00798353 | 122 | 18618 |
| kmer:15 n=2 b=30 c=1.4 e=1 | 1090 | 138 | 3707 | 1663 | 947 | 189 | 0.881523 | 0.00116096 | 0.00947481 | 164 | 18351 |
| kmer:15 n=10 b=30 c=1.4 e=1 | 826.297 | 115 | 3178 | 1292 | 698 | 118 | 0.86618 | 0.000697183 | 0.00308677 | 42 | 17795 |
| kmer:17 n=6 b=68 c=1.0 e=1 | 298.79 | 103 | 1065 | 259 | 104 | 0 | 0.722787 | 0.000486008 | 0.000793173 | 4 | 14803 |
| kmer:19 n=6 b=76 c=1.0 e=0 | 260.616 | 101 | 925 | 237 | 123 | 0 | 0.759234 | 0.000829696 | 0.000873746 | 0 | 15677 |
| kmer:19 n=2 b=38 c=1.2 e=1 | 1381.06 | 167 | 4328 | 2116 | 1180 | 222 | 0.870543 | 0.00114793 | 0.00703647 | 115 | 18081 |
| kmer:19 n=10 b=76 c=1.0 e=0 | 249.823 | 101 | 854 | 223 | 120 | 0 | 0.758002 | 0.000820838 | 0.000839057 | 0 | 15688 |
| kmer:19 n=6 b=76 c=1.4 e=1 | 1133.41 | 141 | 3832 | 1811 | 1075 | 282 | 0.913215 | 0.00113292 | 0.00346373 | 47 | 18792 |
| kmer:15 n=10 b=30 c=1.2 e=1 | 898.277 | 120 | 3414 | 1435 | 784 | 142 | 0.869407 | 0.000705045 | 0.00297579 | 40 | 17865 |
| kmer:17 n=2 b=34 c=2.0 e=0 | 804.73 | 119 | 2952 | 1209 | 699 | 143 | 0.895267 | 0.000985586 | 0.00558137 | 86 | 18453 |
| kmer:15 n=10 b=30 c=2.0 e=1 | 621.882 | 109 | 2487 | 892 | 465 | 52 | 0.848922 | 0.000685109 | 0.00412976 | 59 | 17441 |
| kmer:13 n=2 b=26 c=2.0 e=1 | 519.322 | 105 | 2001 | 630 | 282 | 7 | 0.766659 | 0.00144988 | 0.011447 | 172 | 16288 |
| kmer:17 n=10 b=34 c=1.0 e=0 | 172.935 | 97 | 477 | 78 | 0 | 0 | 0.508415 | 0.000660915 | 0.000791305 | 1 | 10428 |
| kmer:19 n=6 b=38 c=1.0 e=0 | 179.379 | 99 | 504 | 99 | 0 | 0 | 0.546243 | 0.000784262 | 0.000819791 | 0 | 11224 |
| kmer:15 n=2 b=60 c=1.2 e=1 | 1271.66 | 156 | 4138 | 1988 | 1149 | 253 | 0.889978 | 0.00165177 | 0.0111605 | 190 | 18604 |
| kmer:19 n=10 b=38 c=1.4 e=0 | 1049.68 | 125 | 3964 | 1873 | 1126 | 396 | 0.941955 | 0.000833013 | 0.00147558 | 12 | 19366 |
| kmer:15 n=2 b=30 c=1.4 e=0 | 1110.99 | 131 | 3936 | 1847 | 1084 | 315 | 0.912692 | 0.00106551 | 0.00571535 | 92 | 19036 |
| kmer:17 n=6 b=34 c=1.4 e=1 | 1098.64 | 141 | 3731 | 1688 | 940 | 167 | 0.872265 | 0.000738528 | 0.00465811 | 73 | 17916 |
| kmer:17 n=6 b=68 c=1.2 e=1 | 1311.74 | 165 | 4231 | 2056 | 1164 | 238 | 0.880873 | 0.00120307 | 0.00632025 | 97 | 18139 |
| kmer:13 n=2 b=52 c=1.2 e=1 | 672.021 | 109 | 2501 | 908 | 464 | 40 | 0.80261 | 0.0016831 | 0.00913675 | 137 | 17251 |
| kmer:13 n=2 b=52 c=1.0 e=0 | 109.742 | 68 | 289 | 8 | 0 | 0 | 0.275461 | 0.000365297 | 0.000534498 | 1 | 5671 |
| kmer:17 n=6 b=68 c=1.0 e=0 | 274.513 | 102 | 971 | 241 | 111 | 0 | 0.735917 | 0.000682832 | 0.0009018 | 3 | 15122 |

Table S1E - Evaluation of low sequencing depth assembly with soapDeNovo.

| **Parameter Settings** | **Mean** | **Min** | **Max** | **N50** | **N75** | **N90** | **Coverage** | **Error** | **Total Error** | **Unmapped** | **All** |
| --- | --- | --- | --- | --- | --- | --- | --- | --- | --- | --- | --- |
| Kmer: 13 -M 0 | 384.79 | 104 | 1540 | 471 | 273 | 82 | 0.87947 | 0.000472357 | 0.0011617 | 12 | 18176 |
| Kmer: 13 -M 1 | 447.511 | 106 | 1796 | 586 | 342 | 112 | 0.893773 | 0.000646363 | 0.00112631 | 8 | 18491 |
| Kmer: 13 -M 2 | 498.883 | 107 | 2077 | 700 | 404 | 135 | 0.904168 | 0.000782716 | 0.00189791 | 20 | 18723 |
| Kmer: 13 -M 3 | 500.571 | 107 | 2090 | 703 | 406 | 136 | 0.904755 | 0.000772936 | 0.00189871 | 21 | 18736 |
| Kmer: 15 -M 0 | 487.034 | 108 | 1950 | 665 | 392 | 134 | 0.910935 | 0.000535884 | 0.00102203 | 8 | 18772 |
| Kmer: 15 -M 1 | 688.446 | 116 | 2718 | 1051 | 622 | 223 | 0.926615 | 0.000652513 | 0.000949038 | 5 | 19085 |
| Kmer: 15 -M 2 | 921.761 | 123 | 3635 | 1598 | 940 | 338 | 0.937523 | 0.000702016 | 0.00102623 | 6 | 19308 |
| Kmer: 15 -M 3 | 931.618 | 123 | 3681 | 1624 | 957 | 344 | 0.938597 | 0.000712872 | 0.00103654 | 6 | 19331 |
| Kmer: 17 -M 0 | 507.594 | 108 | 2012 | 712 | 425 | 150 | 0.92204 | 0.000629373 | 0.000922506 | 5 | 19011 |
| Kmer: 17 -M 1 | 788.243 | 119 | 3007 | 1243 | 750 | 282 | 0.938112 | 0.000705106 | 0.000839215 | 2 | 19316 |
| Kmer: 17 -M 2 | 1075.43 | 130 | 4096 | 1929 | 1157 | 441 | 0.94794 | 0.000732059 | 0.000950511 | 4 | 19509 |
| Kmer: 17 -M 3 | 1093.6 | 130 | 4181 | 1991 | 1183 | 454 | 0.949321 | 0.000738146 | 0.000951649 | 4 | 19540 |
| Kmer: 19 -M 0 | 501.361 | 108 | 1987 | 706 | 421 | 153 | 0.927751 | 0.000703507 | 0.00100412 | 5 | 19158 |
| Kmer: 19 -M 1 | 801.098 | 120 | 3041 | 1276 | 782 | 292 | 0.944447 | 0.000778647 | 0.000954282 | 3 | 19467 |
| Kmer: 19 -M 2 | 1043.5 | 130 | 3961 | 1851 | 1111 | 424 | 0.952787 | 0.000787422 | 0.000975081 | 3 | 19626 |
| Kmer: 19 -M 3 | 1059.86 | 131 | 4026 | 1897 | 1136 | 437 | 0.954249 | 0.000805661 | 0.000980857 | 3 | 19658 |
| Kmer: 21 -M 0 | 486.973 | 108 | 1921 | 683 | 410 | 154 | 0.930678 | 0.000789377 | 0.00110459 | 6 | 19260 |
| Kmer: 21 -M 1 | 788.043 | 119 | 3017 | 1261 | 780 | 289 | 0.948229 | 0.000869027 | 0.0010005 | 2 | 19577 |
| Kmer: 21 -M 2 | 954.212 | 125 | 3645 | 1644 | 1003 | 379 | 0.954463 | 0.000876887 | 0.00101017 | 2 | 19690 |
| Kmer: 21 -M 3 | 963.704 | 125 | 3669 | 1664 | 1017 | 385 | 0.955538 | 0.000892282 | 0.00102557 | 2 | 19714 |
| Kmer: 23 -M 0 | 473.524 | 107 | 1845 | 662 | 396 | 153 | 0.931956 | 0.000845104 | 0.00122676 | 7 | 19329 |
| Kmer: 23 -M 1 | 750.864 | 118 | 2869 | 1195 | 733 | 273 | 0.949735 | 0.000905108 | 0.00106301 | 3 | 19637 |
| Kmer: 23 -M 2 | 848.963 | 121 | 3232 | 1420 | 865 | 322 | 0.953844 | 0.000919877 | 0.00106097 | 2 | 19704 |
| Kmer: 23 -M 3 | 853.987 | 121 | 3247 | 1427 | 870 | 325 | 0.954361 | 0.000939317 | 0.00115517 | 4 | 19717 |
