# Supplementary material for: LOCAS – A Low Coverage Assembly Tool for Resequencing Projects
Source: PLoS One. 2011 Aug 15;6(8):e23455. doi: 10.1371/journal.pone.0023455 (PMC3156226; doi:10.1371/journal.pone.0023455)
Supplement: Table S2 — Evaluation of homology-guided assembly on simulated data with SUPERLOCAS and VELVET. (DOC) [file pone.0023455.s008.doc]

Table 2A - Evaluation of homology-guided assembly on simulated data with SUPERLOCAS.

| **Parameter Settings** | **Mean** | **Min** | **Max** | **N50** | **N75** | **N90** | **Coverage** | **Error** | **Total Error** | **Unmapped** | **All** |
| --- | --- | --- | --- | --- | --- | --- | --- | --- | --- | --- | --- |
| kmer:19 -L 17 -S 1 | 2330.75 | 500 | 17101 | 2974 | 1716 | 382 | 0.852468 | 0.00232651 | 0.433293 | 1710539 | 2249098 |
| kmer:19 -L 19 -S 1 | 2342.08 | 500 | 17101 | 2986 | 1726 | 383 | 0.852517 | 0.0023325 | 0.423862 | 1645747 | 2249459 |
| kmer:19 -L 21 -S 1 | 2466.42 | 500 | 16905 | 3049 | 1773 | 391 | 0.854089 | 0.00198879 | 0.225333 | 625942 | 2197188 |
| kmer:19 -L 23 -S 1 | 2514.92 | 500 | 16946 | 3068 | 1801 | 396 | 0.85481 | 0.00188842 | 0.0802353 | 189403 | 2179452 |
| kmer:19 -L 25 -S 1 | 2522.17 | 500 | 16946 | 3074 | 1804 | 399 | 0.855518 | 0.00184713 | 0.0604777 | 139102 | 2180193 |
| kmer:19 -L 27 -S 1 | 2534.58 | 500 | 17431 | 3088 | 1812 | 402 | 0.856304 | 0.00179155 | 0.0491413 | 111117 | 2180768 |
| kmer:21 -L 17 -S 1 | 2491.17 | 500 | 17166 | 3046 | 1780 | 392 | 0.854877 | 0.00195305 | 0.130535 | 319917 | 2183083 |
| kmer:21 -L 19 -S 1 | 2487 | 500 | 17166 | 3050 | 1780 | 392 | 0.854941 | 0.00199277 | 0.135632 | 335507 | 2185910 |
| kmer:21 -L 21 -S 1 | 2497.5 | 500 | 17166 | 3056 | 1782 | 392 | 0.854875 | 0.00195581 | 0.13019 | 319098 | 2183138 |
| kmer:21 -L 23 -S 1 | 2524 | 500 | 17287 | 3072 | 1809 | 398 | 0.855411 | 0.00183345 | 0.0655806 | 151840 | 2179396 |
| kmer:21 -L 25 -S 1 | 2532.42 | 500 | 17188 | 3083 | 1808 | 442 | 0.856056 | 0.00179 | 0.0512035 | 115984 | 2179211 |
| kmer:21 -L 27 -S 1 | 2542.58 | 500 | 17189 | 3092 | 1815 | 446 | 0.856578 | 0.00177216 | 0.0443031 | 99251 | 2179700 |

Table S2B - Evaluation of homology-guided assembly on simulated data with VELVET.

| **Parameter Settings** | **Mean** | **Min** | **Max** | **N50** | **N75** | **N90** | **Coverage** | **Error** | **Total Error** | **Unmapped** | **All** |
| --- | --- | --- | --- | --- | --- | --- | --- | --- | --- | --- | --- |
| kmer:17 -exp_cov 17 | 932.833 | 500 | 5034 | 367 | 0 | 0 | 0.496019 | 0.00657794 | 0.905558 | 17633724 | 1851353 |
| kmer:17 -exp_cov 7 | 918.583 | 500 | 4414 | 297 | 0 | 0 | 0.47353 | 0.00641662 | 0.906551 | 17156517 | 1783589 |
| kmer:17 -exp_cov auto | 581.25 | 501 | 874 | 0 | 0 | 0 | 0.00851731 | 0.0012187 | 0.952284 | 568618 | 25613 |
| kmer:19 -exp_cov 17 | 1463 | 501 | 11789 | 2177 | 1280 | 146 | 0.814079 | 0.00566864 | 0.897822 | 27010232 | 3125195 |
| kmer:19 -exp_cov 7 | 1447.92 | 502 | 11911 | 2110 | 1218 | 135 | 0.806099 | 0.00548915 | 0.8976 | 26810127 | 3109346 |
| kmer:19 -exp_cov auto | 868.917 | 500 | 4006 | 176 | 0 | 0 | 0.432637 | 0.000900552 | 0.889833 | 11325556 | 1405708 |
| kmer:21 -exp_cov 17 | 1464.08 | 500 | 12502 | 2221 | 1306 | 187 | 0.825782 | 0.0039348 | 0.896998 | 26616877 | 3097753 |
| kmer:21 -exp_cov 7 | 1455.83 | 500 | 12445 | 2184 | 1275 | 183 | 0.820642 | 0.00365806 | 0.897175 | 26550914 | 3083186 |
| kmer:21 -exp_cov auto | 1059.17 | 500 | 6761 | 696 | 43 | 0 | 0.607963 | 0.0011114 | 0.88395 | 14882691 | 1961838 |
| kmer:23 -exp_cov 17 | 1367.25 | 500 | 11448 | 1854 | 1093 | 0 | 0.813252 | 0.00380335 | 0.895977 | 25824966 | 3038006 |
| kmer:23 -exp_cov 7 | 1359.92 | 500 | 11242 | 1828 | 1071 | 0 | 0.809554 | 0.00358695 | 0.896102 | 25770145 | 3026377 |
| kmer:23 -exp_cov auto | 1064.83 | 500 | 6652 | 667 | 0 | 0 | 0.598013 | 0.00124133 | 0.884599 | 14694516 | 1934311 |
