# Supplementary material for: LOCAS – A Low Coverage Assembly Tool for Resequencing Projects
Source: PLoS One. 2011 Aug 15;6(8):e23455. doi: 10.1371/journal.pone.0023455 (PMC3156226; doi:10.1371/journal.pone.0023455)
Supplement: Table S3 — Evaluation of homology-guided assembly on real world data with LOCAS and VELVET (without utilizing left-over reads). (DOC) [file pone.0023455.s009.doc]

Table S3A – Evaluation of homology-guided assembly on real world data with LOCAS (without utilizing left-over reads).

| **Parameter Settings** | **Mean** | **Min** | **Max** | **N50** | **N75** | **N90** | **Coverage** | **Error** | **Total Error** | **Unmapped** | **All** |
| --- | --- | --- | --- | --- | --- | --- | --- | --- | --- | --- | --- |
| kmer:13 -L 11 -S 4 | 1312 | 500 | 7312 | 1501 | 744 | 0 | 0.777362 | 0.00492953 | 0.00958249 | 17347 | 3692439 |
| kmer:13 -L 13 -S 4 | 1313 | 500 | 7312 | 1508 | 752 | 0 | 0.778618 | 0.00488424 | 0.00935314 | 16804 | 3725040 |
| kmer:13 -L 15 -S 4 | 1353 | 500 | 9266 | 1519 | 732 | 0 | 0.77195 | 0.00444431 | 0.00801188 | 13301 | 3698437 |
| kmer:13 -L 17 -S 4 | 1342 | 500 | 9463 | 1471 | 694 | 0 | 0.761737 | 0.00429263 | 0.00828065 | 14629 | 3637860 |
| kmer:13 -L 19 -S 4 | 1324 | 500 | 7046 | 1401 | 661 | 0 | 0.750992 | 0.00424553 | 0.00768585 | 12406 | 3578348 |
| kmer:13 -L 11 -S 3 | 1299 | 500 | 7312 | 1488 | 746 | 0 | 0.778258 | 0.00484103 | 0.0107247 | 22120 | 3719253 |
| kmer:13 -L 11 -S 5 | 1316 | 500 | 7312 | 1518 | 744 | 0 | 0.775158 | 0.00488811 | 0.00970901 | 18067 | 3711252 |
| kmer:13 -L 13 -S 3 | 1302 | 500 | 7312 | 1488 | 748 | 0 | 0.778408 | 0.00482619 | 0.0105579 | 21561 | 3721987 |
| kmer:13 -L 13 -S 5 | 1318 | 500 | 7312 | 1517 | 747 | 0 | 0.77544 | 0.00487431 | 0.00969151 | 18067 | 3714170 |
| kmer:13 -L 15 -S 3 | 1343 | 500 | 9266 | 1501 | 730 | 0 | 0.77345 | 0.00453375 | 0.00920431 | 17449 | 3701568 |
| kmer:13 -L 15 -S 5 | 1357 | 500 | 9266 | 1526 | 726 | 0 | 0.76953 | 0.00450161 | 0.0087218 | 15702 | 3688235 |
| kmer:13 -L 17 -S 3 | 1333 | 500 | 9463 | 1454 | 699 | 0 | 0.764372 | 0.00437628 | 0.00885896 | 16490 | 3646018 |
| kmer:13 -L 17 -S 5 | 1346 | 500 | 9463 | 1474 | 687 | 0 | 0.75997 | 0.0043102 | 0.0096489 | 19552 | 3626977 |
| kmer:13 -L 19 -S 3 | 1316 | 500 | 7046 | 1391 | 670 | 0 | 0.75434 | 0.00437027 | 0.00840279 | 14601 | 3590392 |
| kmer:13 -L 19 -S 5 | 1326 | 500 | 7046 | 1401 | 654 | 0 | 0.750282 | 0.00430952 | 0.00911777 | 17320 | 3569304 |
| kmer:13 -L 21 -S 3 | 1303 | 500 | 7046 | 1346 | 644 | 0 | 0.745886 | 0.00426442 | 0.00617173 | 6792 | 3539052 |
| kmer:13 -L 21 -S 4 | 1311 | 500 | 7046 | 1357 | 638 | 0 | 0.742303 | 0.0041246 | 0.00654625 | 8593 | 3525187 |
| kmer:13 -L 21 -S 5 | 1313 | 500 | 7046 | 1360 | 627 | 0 | 0.741471 | 0.0042235 | 0.00817844 | 14027 | 3517698 |
| kmer:13 -L 23 -S 3 | 1277 | 500 | 6285 | 1272 | 612 | 0 | 0.737224 | 0.00422341 | 0.00652912 | 8089 | 3485338 |
| kmer:13 -L 23 -S 4 | 1284 | 500 | 6285 | 1272 | 612 | 0 | 0.734849 | 0.00413187 | 0.00610858 | 6915 | 3476874 |
| kmer:13 -L 23 -S 5 | 1287 | 500 | 6285 | 1280 | 605 | 0 | 0.733268 | 0.00420927 | 0.00788639 | 12853 | 3467826 |
| kmer:13 -L 25 -S 3 | 1255 | 500 | 6285 | 1221 | 581 | 0 | 0.726381 | 0.00416637 | 0.00650812 | 8046 | 3413525 |
| kmer:13 -L 25 -S 4 | 1260 | 500 | 6285 | 1218 | 578 | 0 | 0.725407 | 0.00414883 | 0.00616081 | 6908 | 3412288 |
| kmer:13 -L 25 -S 5 | 1263 | 500 | 6285 | 1222 | 573 | 0 | 0.72326 | 0.00416852 | 0.00750086 | 11418 | 3400725 |
| kmer:13 -L 11 -S 1 | 1275 | 500 | 7194 | 1438 | 724 | 0 | 0.776196 | 0.00487434 | 0.00948872 | 17165 | 3684600 |
| kmer:13 -L 11 -S 2 | 1292 | 500 | 7194 | 1481 | 744 | 0 | 0.778984 | 0.00481438 | 0.00987148 | 18992 | 3718443 |
| kmer:13 -L 13 -S 1 | 1276 | 500 | 7194 | 1438 | 724 | 0 | 0.776237 | 0.00482473 | 0.00968736 | 18099 | 3686006 |
| kmer:13 -L 13 -S 2 | 1295 | 500 | 7194 | 1481 | 747 | 0 | 0.779124 | 0.00483726 | 0.00986041 | 18881 | 3721735 |
| kmer:13 -L 15 -S 1 | 1314 | 500 | 9148 | 1449 | 722 | 0 | 0.770026 | 0.00450158 | 0.00828673 | 14023 | 3674042 |
| kmer:13 -L 15 -S 2 | 1337 | 500 | 9148 | 1488 | 741 | 0 | 0.774379 | 0.0045074 | 0.00877563 | 15952 | 3704578 |
| kmer:13 -L 17 -S 1 | 1310 | 500 | 9463 | 1417 | 685 | 0 | 0.762468 | 0.00430101 | 0.00793577 | 13277 | 3623800 |
| kmer:13 -L 17 -S 2 | 1327 | 500 | 9463 | 1440 | 708 | 0 | 0.764997 | 0.00437361 | 0.00826546 | 14315 | 3647791 |
| kmer:13 -L 19 -S 1 | 1295 | 500 | 7046 | 1358 | 649 | 0 | 0.750828 | 0.00426173 | 0.00670038 | 8753 | 3565223 |
| kmer:13 -L 19 -S 2 | 1312 | 500 | 7046 | 1383 | 676 | 0 | 0.754754 | 0.00436063 | 0.00682609 | 8924 | 3594894 |
| kmer:13 -L 21 -S 1 | 1279 | 500 | 7046 | 1302 | 626 | 0 | 0.741875 | 0.00407003 | 0.00572688 | 5845 | 3507588 |
| kmer:13 -L 21 -S 2 | 1296 | 500 | 7046 | 1338 | 648 | 0 | 0.746407 | 0.00420205 | 0.0057891 | 5650 | 3539462 |
| kmer:13 -L 23 -S 1 | 1255 | 500 | 6285 | 1247 | 592 | 0 | 0.732259 | 0.00404349 | 0.00566676 | 5635 | 3451717 |
| kmer:13 -L 23 -S 2 | 1273 | 500 | 6285 | 1257 | 614 | 0 | 0.73603 | 0.00419771 | 0.0059795 | 6214 | 3466652 |
| kmer:13 -L 25 -S 1 | 1231 | 500 | 6285 | 1177 | 564 | 0 | 0.72049 | 0.00398823 | 0.0056544 | 5656 | 3375428 |
| kmer:13 -L 25 -S 2 | 1249 | 500 | 6285 | 1210 | 582 | 0 | 0.725835 | 0.00414653 | 0.00571945 | 5395 | 3410319 |

Table S3B – Evaluation of homology-guided assembly on real world data with VELVET (without utilizing left-over reads).

**-ins_length 200 -ins_length_sd 20 -scaffolding no**

| **Parameter Settings** | **Mean** | **Min** | **Max** | **N50** | **N75** | **N90** | **Coverage** | **Error** | **Total Error** | **Unmapped** | **All** |
| --- | --- | --- | --- | --- | --- | --- | --- | --- | --- | --- | --- |
| kmer:11 -exp_cov 3 | 737 | 602 | 873 | 0 | 0 | 0 | 0.000549257 | 0.00610169 | 0.00610169 | 0 | 1475 |
| kmer:11 -exp_cov 5 | 592 | 505 | 873 | 0 | 0 | 0 | 0.00467967 | 0.0168647 | 0.0949188 | 1125 | 13045 |
| kmer:11 -exp_cov 7 | 635 | 500 | 1278 | 0 | 0 | 0 | 0.0185906 | 0.0120378 | 0.0532132 | 2323 | 53415 |
| kmer:11 -exp_cov 9 | 643 | 500 | 1278 | 0 | 0 | 0 | 0.0428968 | 0.00875858 | 0.0441525 | 4697 | 126847 |
| kmer:11 -exp_cov 11 | 692 | 500 | 2507 | 0 | 0 | 0 | 0.0789932 | 0.00814128 | 0.0325501 | 6102 | 241854 |
| kmer:11 -exp_cov 13 | 705 | 500 | 2507 | 0 | 0 | 0 | 0.108582 | 0.00770808 | 0.0336219 | 8972 | 334584 |
| kmer:11 -exp_cov 15 | 720 | 500 | 2507 | 0 | 0 | 0 | 0.132746 | 0.0070234 | 0.0293147 | 9541 | 415468 |
| kmer:11 -exp_cov 17 | 726 | 500 | 2507 | 0 | 0 | 0 | 0.144515 | 0.00684625 | 0.0322745 | 12040 | 458207 |
| kmer:11 -exp_cov 19 | 732 | 500 | 2507 | 0 | 0 | 0 | 0.15235 | 0.0070263 | 0.0318239 | 12547 | 489874 |
| kmer:11 -exp_cov 21 | 736 | 500 | 2507 | 0 | 0 | 0 | 0.155852 | 0.00715524 | 0.0313189 | 12547 | 502988 |
| kmer:11 -exp_cov 23 | 737 | 500 | 2507 | 0 | 0 | 0 | 0.157612 | 0.0071227 | 0.031018 | 12547 | 508796 |
| kmer:13 -exp_cov 3 | 682 | 500 | 1885 | 0 | 0 | 0 | 0.260833 | 0.00322693 | 0.0108927 | 6451 | 832369 |
| kmer:13 -exp_cov 5 | 722 | 500 | 2082 | 0 | 0 | 0 | 0.379329 | 0.00468802 | 0.0215565 | 21653 | 1255967 |
| kmer:13 -exp_cov 7 | 836 | 500 | 3635 | 529 | 0 | 0 | 0.523924 | 0.00564608 | 0.0261868 | 40441 | 1917260 |
| kmer:13 -exp_cov 9 | 986 | 500 | 5647 | 737 | 0 | 0 | 0.623347 | 0.00611657 | 0.0349251 | 75387 | 2525435 |
| kmer:13 -exp_cov 11 | 1138 | 500 | 6106 | 1002 | 0 | 0 | 0.682931 | 0.00671434 | 0.0349666 | 85979 | 2936849 |
| kmer:13 -exp_cov 13 | 1254 | 500 | 7563 | 1198 | 522 | 0 | 0.707654 | 0.00693122 | 0.0392904 | 106152 | 3151537 |
| kmer:13 -exp_cov 15 | 1321 | 500 | 7563 | 1309 | 550 | 0 | 0.714542 | 0.0072071 | 0.0393136 | 108551 | 3248046 |
| kmer:13 -exp_cov 17 | 1339 | 500 | 7563 | 1340 | 564 | 0 | 0.718266 | 0.00724114 | 0.0411456 | 116052 | 3282079 |
| kmer:13 -exp_cov 19 | 1355 | 500 | 8518 | 1357 | 578 | 0 | 0.720095 | 0.00730789 | 0.0414968 | 117742 | 3300954 |
| kmer:13 -exp_cov 21 | 1354 | 500 | 8518 | 1359 | 586 | 0 | 0.721978 | 0.00724782 | 0.041984 | 119959 | 3308443 |
| kmer:13 -exp_cov 23 | 1354 | 500 | 8518 | 1361 | 589 | 0 | 0.72332 | 0.00725723 | 0.0419514 | 119959 | 3312560 |
| kmer:15 -exp_cov 3 | 855 | 500 | 4167 | 550 | 0 | 0 | 0.537818 | 0.00311812 | 0.00725428 | 8430 | 2023337 |
| kmer:15 -exp_cov 5 | 877 | 500 | 4167 | 642 | 0 | 0 | 0.598817 | 0.00371195 | 0.0128878 | 21424 | 2304721 |
| kmer:15 -exp_cov 7 | 1000 | 500 | 5424 | 834 | 0 | 0 | 0.670457 | 0.00479145 | 0.0158779 | 31129 | 2763257 |
| kmer:15 -exp_cov 9 | 1175 | 500 | 9833 | 1121 | 559 | 0 | 0.720799 | 0.005485 | 0.015345 | 31642 | 3159891 |
| kmer:15 -exp_cov 11 | 1311 | 500 | 9833 | 1326 | 628 | 0 | 0.74122 | 0.00586209 | 0.0155385 | 33191 | 3376780 |
| kmer:15 -exp_cov 13 | 1401 | 500 | 9833 | 1510 | 669 | 0 | 0.750695 | 0.00597809 | 0.017854 | 42092 | 3481043 |
| kmer:15 -exp_cov 15 | 1441 | 500 | 9833 | 1543 | 687 | 0 | 0.754511 | 0.0061555 | 0.0198706 | 49314 | 3524163 |
| kmer:15 -exp_cov 17 | 1457 | 500 | 11455 | 1575 | 696 | 0 | 0.755969 | 0.00616665 | 0.0200156 | 50084 | 3544063 |
| kmer:15 -exp_cov 19 | 1459 | 500 | 11455 | 1583 | 711 | 0 | 0.758022 | 0.00634489 | 0.0199259 | 49321 | 3559244 |
| kmer:15 -exp_cov 21 | 1462 | 500 | 11455 | 1595 | 715 | 0 | 0.759152 | 0.00650993 | 0.0200786 | 49321 | 3561941 |
| kmer:15 -exp_cov 23 | 1463 | 500 | 11455 | 1606 | 717 | 0 | 0.759556 | 0.00645307 | 0.0200134 | 49321 | 3564352 |
| kmer:17 -exp_cov 3 | 887 | 500 | 4117 | 582 | 0 | 0 | 0.557884 | 0.00309605 | 0.00364001 | 1158 | 2121091 |
| kmer:17 -exp_cov 5 | 905 | 500 | 4117 | 674 | 0 | 0 | 0.616183 | 0.00373046 | 0.0071816 | 8350 | 2402117 |
| kmer:17 -exp_cov 7 | 1038 | 500 | 4683 | 896 | 0 | 0 | 0.683068 | 0.00443637 | 0.0112802 | 19647 | 2838359 |
| kmer:17 -exp_cov 9 | 1199 | 500 | 8419 | 1158 | 567 | 0 | 0.722684 | 0.00497519 | 0.0116263 | 21517 | 3197464 |
| kmer:17 -exp_cov 11 | 1317 | 500 | 9194 | 1363 | 629 | 0 | 0.73997 | 0.00522006 | 0.0109884 | 19726 | 3382146 |
| kmer:17 -exp_cov 13 | 1392 | 500 | 9458 | 1476 | 656 | 0 | 0.747357 | 0.00543996 | 0.01116 | 20143 | 3482193 |
| kmer:17 -exp_cov 15 | 1421 | 500 | 9458 | 1514 | 667 | 0 | 0.749665 | 0.00554672 | 0.0129999 | 26561 | 3517394 |
| kmer:17 -exp_cov 17 | 1429 | 500 | 9458 | 1527 | 674 | 0 | 0.752174 | 0.00558917 | 0.0132373 | 27373 | 3531649 |
| kmer:17 -exp_cov 19 | 1435 | 500 | 9458 | 1543 | 685 | 0 | 0.753917 | 0.00564669 | 0.0135878 | 28514 | 3541895 |
| kmer:17 -exp_cov 21 | 1437 | 500 | 9458 | 1553 | 692 | 0 | 0.754096 | 0.0056735 | 0.0136108 | 28514 | 3543493 |
| kmer:17 -exp_cov 23 | 1436 | 500 | 9458 | 1553 | 704 | 0 | 0.754891 | 0.0056932 | 0.0136723 | 28693 | 3546861 |
| kmer:19 -exp_cov 3 | 904 | 500 | 4435 | 597 | 0 | 0 | 0.565362 | 0.00311035 | 0.00432148 | 2630 | 2162138 |
| kmer:19 -exp_cov 5 | 922 | 500 | 4435 | 697 | 0 | 0 | 0.626778 | 0.0035687 | 0.00708879 | 8757 | 2470088 |
| kmer:19 -exp_cov 7 | 1059 | 500 | 6619 | 928 | 0 | 0 | 0.68909 | 0.00432238 | 0.00799594 | 10741 | 2900487 |
| kmer:19 -exp_cov 9 | 1219 | 500 | 8421 | 1173 | 575 | 0 | 0.722497 | 0.00488906 | 0.00805816 | 10313 | 3228021 |
| kmer:19 -exp_cov 11 | 1328 | 500 | 8421 | 1365 | 623 | 0 | 0.735934 | 0.0052144 | 0.00841642 | 10957 | 3393104 |
| kmer:19 -exp_cov 13 | 1384 | 500 | 8421 | 1427 | 654 | 0 | 0.742657 | 0.00533689 | 0.00925887 | 13706 | 3462314 |
| kmer:19 -exp_cov 15 | 1406 | 500 | 8421 | 1447 | 666 | 0 | 0.745817 | 0.00540139 | 0.00948705 | 14414 | 3494473 |
| kmer:19 -exp_cov 17 | 1414 | 500 | 8421 | 1470 | 673 | 0 | 0.746845 | 0.00543397 | 0.00947513 | 14302 | 3505543 |
| kmer:19 -exp_cov 19 | 1416 | 500 | 8421 | 1478 | 681 | 0 | 0.746898 | 0.00559817 | 0.00944453 | 13627 | 3509364 |
| kmer:19 -exp_cov 21 | 1420 | 500 | 8421 | 1480 | 684 | 0 | 0.747429 | 0.0055955 | 0.00943927 | 13627 | 3511753 |
| kmer:19 -exp_cov 23 | 1420 | 500 | 8421 | 1482 | 687 | 0 | 0.748159 | 0.00562112 | 0.00946226 | 13627 | 3514070 |
| kmer:21 -exp_cov 3 | 900 | 500 | 4612 | 602 | 0 | 0 | 0.572872 | 0.00322384 | 0.00466156 | 3144 | 2176599 |
| kmer:21 -exp_cov 5 | 922 | 500 | 4612 | 694 | 0 | 0 | 0.62871 | 0.00352128 | 0.00647335 | 7315 | 2461887 |
| kmer:21 -exp_cov 7 | 1053 | 500 | 6618 | 916 | 0 | 0 | 0.688573 | 0.00426654 | 0.00990835 | 16533 | 2901411 |
| kmer:21 -exp_cov 9 | 1204 | 500 | 6761 | 1145 | 562 | 0 | 0.71965 | 0.00472558 | 0.00940517 | 15170 | 3211246 |
| kmer:21 -exp_cov 11 | 1305 | 500 | 6761 | 1312 | 605 | 0 | 0.730192 | 0.00492489 | 0.00939543 | 15151 | 3357233 |
| kmer:21 -exp_cov 13 | 1343 | 500 | 6957 | 1353 | 615 | 0 | 0.734025 | 0.00515165 | 0.0105271 | 18559 | 3416189 |
| kmer:21 -exp_cov 15 | 1358 | 500 | 6957 | 1376 | 625 | 0 | 0.736349 | 0.00520235 | 0.0105293 | 18529 | 3441712 |
| kmer:21 -exp_cov 17 | 1360 | 500 | 6957 | 1378 | 628 | 0 | 0.737648 | 0.00531539 | 0.0113236 | 20952 | 3447727 |
| kmer:21 -exp_cov 19 | 1363 | 500 | 6957 | 1384 | 630 | 0 | 0.737548 | 0.00534237 | 0.0113472 | 20952 | 3449594 |
| kmer:21 -exp_cov 21 | 1365 | 500 | 6957 | 1384 | 630 | 0 | 0.737382 | 0.00535159 | 0.0113557 | 20952 | 3450004 |
| kmer:21 -exp_cov 23 | 1365 | 500 | 6957 | 1385 | 630 | 0 | 0.737334 | 0.00534842 | 0.0115607 | 21676 | 3448869 |
| kmer:23 -exp_cov 3 | 893 | 500 | 5419 | 594 | 0 | 0 | 0.565854 | 0.00291522 | 0.0050449 | 4625 | 2160732 |
| kmer:23 -exp_cov 5 | 920 | 500 | 5419 | 694 | 0 | 0 | 0.62378 | 0.00340794 | 0.00638834 | 7389 | 2463362 |
| kmer:23 -exp_cov 7 | 1057 | 500 | 6619 | 913 | 0 | 0 | 0.682054 | 0.00408132 | 0.00922508 | 15028 | 2894650 |
| kmer:23 -exp_cov 9 | 1201 | 500 | 6619 | 1120 | 529 | 0 | 0.709706 | 0.00442108 | 0.00886183 | 14262 | 3183158 |
| kmer:23 -exp_cov 11 | 1269 | 500 | 6836 | 1213 | 562 | 0 | 0.719478 | 0.00462502 | 0.00956077 | 16423 | 3295550 |
| kmer:23 -exp_cov 13 | 1300 | 500 | 6836 | 1254 | 574 | 0 | 0.722176 | 0.00482885 | 0.00968934 | 16423 | 3346137 |
| kmer:23 -exp_cov 15 | 1312 | 500 | 6836 | 1273 | 584 | 0 | 0.724761 | 0.00503737 | 0.00966752 | 15756 | 3370013 |
| kmer:23 -exp_cov 17 | 1315 | 500 | 6836 | 1275 | 585 | 0 | 0.724817 | 0.00508824 | 0.00992654 | 16497 | 3375822 |
| kmer:23 -exp_cov 19 | 1316 | 500 | 6836 | 1275 | 584 | 0 | 0.724463 | 0.00505368 | 0.0104287 | 18322 | 3373188 |
| kmer:23 -exp_cov 21 | 1316 | 500 | 6836 | 1274 | 585 | 0 | 0.725036 | 0.00508967 | 0.0116469 | 22371 | 3371930 |
| kmer:23 -exp_cov 23 | 1316 | 500 | 6836 | 1274 | 583 | 0 | 0.72446 | 0.00509293 | 0.011652 | 22371 | 3370947 |
| kmer:25 -exp_cov 3 | 897 | 500 | 5421 | 591 | 0 | 0 | 0.565139 | 0.00295546 | 0.0038929 | 2044 | 2171915 |
| kmer:25 -exp_cov 5 | 929 | 500 | 5421 | 692 | 0 | 0 | 0.622104 | 0.00339364 | 0.00614317 | 6860 | 2479641 |
| kmer:25 -exp_cov 7 | 1066 | 500 | 5812 | 913 | 0 | 0 | 0.678177 | 0.00395847 | 0.00695147 | 8788 | 2915773 |
| kmer:25 -exp_cov 9 | 1184 | 500 | 5857 | 1078 | 510 | 0 | 0.702114 | 0.00424866 | 0.00767758 | 10908 | 3156758 |
| kmer:25 -exp_cov 11 | 1241 | 500 | 6836 | 1142 | 526 | 0 | 0.707634 | 0.00439449 | 0.00768439 | 10790 | 3254530 |
| kmer:25 -exp_cov 13 | 1263 | 500 | 6836 | 1164 | 534 | 0 | 0.709234 | 0.00445998 | 0.00884669 | 14529 | 3282748 |
| kmer:25 -exp_cov 15 | 1268 | 500 | 6836 | 1168 | 538 | 0 | 0.710232 | 0.00462931 | 0.00871837 | 13587 | 3293796 |
| kmer:25 -exp_cov 17 | 1269 | 500 | 6836 | 1174 | 540 | 0 | 0.71132 | 0.0046209 | 0.00870181 | 13587 | 3300437 |
| kmer:25 -exp_cov 19 | 1270 | 500 | 6836 | 1175 | 543 | 0 | 0.711776 | 0.00472245 | 0.00880089 | 13587 | 3302101 |
| kmer:25 -exp_cov 21 | 1269 | 500 | 6836 | 1175 | 544 | 0 | 0.712095 | 0.00474031 | 0.00881764 | 13587 | 3302948 |
| kmer:25 -exp_cov 23 | 1270 | 500 | 6836 | 1177 | 544 | 0 | 0.711984 | 0.00475309 | 0.00883197 | 13587 | 3301642 |
