# Supplementary material for: LOCAS – A Low Coverage Assembly Tool for Resequencing Projects
Source: PLoS One. 2011 Aug 15;6(8):e23455. doi: 10.1371/journal.pone.0023455 (PMC3156226; doi:10.1371/journal.pone.0023455)
Supplement: Table S4 — Evaluation of homology-guided assembly on real world data with SUPERLOCAS and VELVET (utilizing left-over reads). (DOC) [file pone.0023455.s010.doc]

Table S4A – Evaluation of homology-guided assembly on real world data with LOCAS (utilizing left-over reads).

-Lt 15 -St 4 -P kmer 13 -K(Kmerg) 21

| **Parameter Settings** | **N50** |
| --- | --- |
| kmer:21 -Llo(Lm) 19 -Slo(Sm) 0 -DR 19 150 | 1514 |
| kmer:21 -Llo(Lm) 19 -Slo(Sm) 0 -DR 21 150 | 1514 |
| kmer:21 -Llo(Lm) 19 -Slo(Sm) 0 -DR 17 300 | 1514 |
| kmer:21 -Llo(Lm) 19 -Slo(Sm) 0 -DR 17 500 | 1513 |
| kmer:21 -Llo(Lm) 21 -Slo(Sm) 0 -DR 19 150 | 1514 |
| kmer:21 -Llo(Lm) 21 -Slo(Sm) 0 -DR 21 150 | 1514 |
| kmer:21 -Llo(Lm) 21 -Slo(Sm) 0 -DR 17 300 | 1514 |
| kmer:21 -Llo(Lm) 21 -Slo(Sm) 0 -DR 17 500 | 1513 |
| kmer:21 -Llo(Lm) 23 -Slo(Sm) 0 -DR 19 150 | 1514 |
| kmer:21 -Llo(Lm) 23 -Slo(Sm) 0 -DR 21 150 | 1514 |
| kmer:21 -Llo(Lm) 23 -Slo(Sm) 0 -DR 17 300 | 1514 |
| kmer:21 -Llo(Lm) 23 -Slo(Sm) 0 -DR 17 500 | 1513 |
| kmer:21 -Llo(Lm) 19 -Slo(Sm) 0 -DR 17 150 | 1514 |
| kmer:21 -Llo(Lm) 21 -Slo(Sm) 0 -DR 17 150 | 1514 |
| kmer:21 -Llo(Lm) 23 -Slo(Sm) 0 -DR 17 150 | 1514 |

Table S4B – Evaluation of homology-guided assembly on real world data with VELVET (utilizing left-over reads).

-ins_length 200 -ins_length_sd 20 -scaffolding no

| **Parameter Settings** | **N50** |
| --- | --- |
| kmer:19 -exp_cov 27 | 1435 |
| kmer:21 -exp_cov 17 | 1379 |
| kmer:21 -exp_cov 27 | 1379 |
| kmer:23 -exp_cov 17 | 1285 |
| kmer:23 -exp_cov 27 | 1293 |
| kmer:21 -exp_cov 7 | 935 |
| kmer:23 -exp_cov 7 | 935 |
| kmer:19 -exp_cov 17 | 1427 |
| kmer:19 -exp_cov 27 | 1435 |
| kmer:19 -exp_cov 7 | 901 |
| kmer:19 -exp_cov auto | 1110 |
| kmer:21 -exp_cov 17 | 1379 |
| kmer:21 -exp_cov 7 | 936 |
| kmer:21 -exp_cov auto | 1196 |
| kmer:23 -exp_cov auto | 1160 |
