# Supplementary material for: LOCAS – A Low Coverage Assembly Tool for Resequencing Projects
Source: PLoS One. 2011 Aug 15;6(8):e23455. doi: 10.1371/journal.pone.0023455 (PMC3156226; doi:10.1371/journal.pone.0023455)
Supplement: Text S1 — Supplementary Results and Methods. Results of further assemblies for the first chromosome of A. thaliana at a sequencing depth of 5× and for the fourth chromosome of A. thaliana at a sequencing depth of 7× are shown. In addition, the usage of LOCAS and SUPERLOCAS is described. In the Section Methods the read simulation method, the simulation of a homology-guided assembly and the assembly analysis are described more precisely. (DOC) [file pone.0023455.s011.doc]

**Supplementary Material**

**Results**

**Evaluation of Assembly for the First Chromosome of A. thaliana at a Sequencing Depth of 5x**

This study was performed in the same manner as the study “*Evaluation of Low Sequencing Depth Assembly*“ with the only alteration that Illumina reads were simulated at a lower sequencing depth of 5x. As in the other study, reads were simulated with the first chromosome of *A. thaliana* Col-0 as reference sequence. The reads were then assigned to their origin positions and partitioned into blocks of a length of 10 kb. We ran the assemblers LOCAS, ABySS, EULER-SR, VELVET and SOAPdenovo using a wide range of parameter settings to show the achievable avgN50 and avgERR values with the respective assembler. Assemblies of the blocks were performed separately.

The results are shown in Figure 1. LOCAS performed best with an avgN50 size of 1,204 bp and an avgERR of 1.4% for the best run. VELVET showed a maximum avgN50 of 1,199 bp with an avgERR of 2%. For smaller avgErr sizes than 2%, the best avgN50 size was 1,170 bp for VELVET. EULER-SR showed an avgERR that ranged between 8% and 14,4%. The avgERR values were low for SOAPdenovo while the maximum avgN50 size was 743 bp. ABySS showed the lowest avgN50 sizes in the comparison.

**Evaluation of Assembly for the Fourth Chromosome of A. thaliana at a Sequencing Depth of 7x**

This study is similar to the study “*Evaluation of Low Sequencing Depth Assembly*“ except that an other reference sequence is used. Here, the forth chromosome of *A. thaliana* is used to simulate Illumina reads. As in the other study, reads were assigned to their origin positions and partitioned into blocks of a length of 10 kb. The assemblies were performed with the assemblers LOCAS, ABySS, EULER-SR, VELVET and SOAPdenovo. Each block was assembled separately and the assemblers were run with a wide range of parameter settings.

The results are shown in Figure 2. LOCAS performed best with an avgN50 size of 4,384 bp and an avgERR of 1.4% for its best run. At the same range of avgERR, VELVET showed an avgN50 size of 3,887 bp. VELVET showed a maximum avgN50 size of 4,043 bp with an avgERR of 2.5%. The avgERR of EULER-SR ranged between 2% and 7.8 % and also its maximum avgN50 size was the lowest with 1,395 bp. The maximum avgN50 sizes of SOAPdenovo and ABySS were 1,959 bp and 1,898 bp, respectively. Both assemblers again showed a low avgERR.

**Usage**

We have implemented LOCAS and SUPERLOCAS as stand-alone tools in C++ using the SeqAn library [1]. LOCAS accepts one or several fasta or fastq files containing the original reads from one library. Several optional parameters allow the fine-tuning of LOCAS, e.g. kmer size, minimum overlap, maximally allowed substitutions in overlaps and the minimum contig size. SUPERLOCAS performs multiple local assemblies consecutively using only one left-over graph and therefore requires separate fasta or fastq files with reads for each local assembly plus one file with left-over reads. All input files have to be listed in an additional input configuration file. A second configuration file listing all output folders is required.

Both assemblers allow the definition of different alignment constraints between reads depending on their alignment position to the reference genome. In addition, SUPERLOCAS provides the possibility of defining different alignment constraints where both reads are left-over reads and also where only one read is a left-over read.

**Methods**

**Read Simulation**

For the two simulation studies, we generated read sets with sequencing depth of 7.5x using METASIM [2]. We used an error model for Illumina reads with a read length of 80 bp as estimated by resequencing the reference strain Col-0 followed by aligning the reads against the reference sequence [F Ott, pers. comm.]. Paired end reads were generated with an insert size of 300 bp or 200 bp, and a standard deviation of 30 bp or 20 bp for the first and second simulation study, respectively.

**Simulation of a Homology-Guided Assembly**

We simulated the genome of a wild strain of *Arabidopsis thaliana* by randomly introducing insertions and deletions into its reference genome sequence. The frequency of SNPs, deletions and small insertions was modeled according to a set of polymorphism from *A. thaliana* strains produced by the Arabidopsis thaliana 1001 genomes project ([www.1001genomes.org](http://www.1001genomes.org/)). This synthetic strain was used to simulate paired-end Illumina reads. In the next step, an alignment of the reads to the reference genome Col-0 was performed. A read is deemed alignable if the alignment contains a maximum of six mismatches and three gaps. Reads aligning into a long insertion or reads spanning a position with a long deletion in the genome are usually not alignable. Next, the chromosomes are partitioned for the local assembly step. (This can be done using a static region size or dynamically using regions with zero coverage or repetitive regions as natural borders.) We used a fixed block length of 25 kb. Almost 2.5% of the reads were non-alignable and were defined as left-over reads. The left-over reads of all chromosomes are pooled since they could not be separated in a real resequencing project.

**Assembly Analysis**

For the simulated datasets, contigs with a minimal length of 100 bp (500 bp if left-over reads were incorporated) were aligned to the the original sequence of the input reads. If the identity was higher or equal to 90%, the contig was considered for further analysis. The N50 and N90 size (analogous to the N50 size), and the mean, minimal and maximal contig size were calculated for each local assembly. The average of all measures over all local assemblies was determined. Next, we calculated the average coverage of the origin block sequences with all aligned contigs. The average total length of aligned and all non-alignable contigs was also determined. If left-over reads were incorporated, we calculated the measures not for each single block but for the pooled contigs of all 100 sequential blocks, called the *100-block*. Thus, contigs are also considered in the analysis if they span two blocks with the help of left-over reads.

For real data from the *A. thaliana* Ler-1 strain, the original genome sequence was not available and thus the performance analysis differed in some points from the analysis of simulated data. As a proxy for the original sequence, we used the reference sequence. Assembled contigs of all blocks were pooled and then aligned against the whole reference genome. During normal assembly mode, the performance was evaluated as described above. If left-over reads were incorporated, the error rate was calculated by considering only the contigs that had a minimum similarity of 75% with the sequence of their 100-block. All non-alignable contigs were not considered for analysis since these contigs do not have to be erroneous and can belong to other 100-blocks consisting only of left-over reads.

1. Doring A, Weese D, Rausch T, Reinert K: **SeqAn an efficient, generic C++ library for sequence analysis.** *BMC Bioinformatics* 2008, **9:**11.

2. Richter DC, Ott F, Auch AF, Schmid R, Huson DH: **MetaSim: a sequencing simulator for genomics and metagenomics.** *PLoS One* 2008, **3:**e3373.
